# The Fear Reduction Exercised Early (FREE) approach to management of low back pain in general practice: A pragmatic cluster-randomised controlled trial

**DOI:** 10.1371/journal.pmed.1002897

**Published:** 2019-09-09

**Authors:** Ben Darlow, James Stanley, Sarah Dean, J. Haxby Abbott, Sue Garrett, Ross Wilson, Fiona Mathieson, Anthony Dowell

**Affiliations:** 1 Department of Primary Health Care and General Practice, University of Otago, Wellington, New Zealand; 2 Biostatistical Group, University of Otago, Wellington, New Zealand; 3 University of Exeter Medical School, College of Medicine and Health, University of Exeter, Exeter, United Kingdom; 4 Department of Surgical Sciences, University of Otago, Dunedin, New Zealand; 5 Department of Psychological Medicine, University of Otago, Wellington, New Zealand; The University of Sydney, AUSTRALIA

## Abstract

**Background:**

Effective and cost-effective primary care treatments for low back pain (LBP) are required to reduce the burden of the world’s most disabling condition. This study aimed to compare the clinical effectiveness and cost-effectiveness of the Fear Reduction Exercised Early (FREE) approach to LBP (intervention) with usual general practitioner (GP) care (control).

**Methods and findings:**

This pragmatic, cluster-randomised controlled trial with process evaluation and parallel economic evaluation was conducted in the Hutt Valley, New Zealand. Eight general practices were randomly assigned (stratified by practice size) with a 1:1 ratio to intervention (4 practices; 34 GPs) or control group (4 practices; 29 GPs). Adults presenting to these GPs with LBP as their primary complaint were recruited. GPs in the intervention practices were trained in the FREE approach, and patients presenting to these practices received care based on the FREE approach. The FREE approach restructures LBP consultations to prioritise early identification and management of barriers to recovery. GPs in control practices did not receive specific training for this study, and patients presenting to these practices received usual care. Between 23 September 2016 and 31 July 2017, 140 eligible patients presented to intervention practices (126 enrolled) and 110 eligible patients presented to control practices (100 enrolled). Patient mean age was 46.1 years (SD 14.4), and 46% were female. The duration of LBP was less than 6 weeks in 88% of patients. Primary outcome was change from baseline in patient participant Roland Morris Disability Questionnaire (RMDQ) score at 6 months. Secondary patient outcomes included pain, satisfaction, and psychosocial indices. GP outcomes included attitudes, knowledge, confidence, and GP LBP management behaviour. There was active and passive surveillance of potential harms. Patients and outcome assessors were blind to group assignment. Analysis followed intention-to-treat principles. A total of 122 (97%) patients from 32 GPs in the intervention group and 99 (99%) patients from 25 GPs in the control group were included in the primary outcome analysis. At 6 months, the groups did not significantly differ on the primary outcome (adjusted mean RMDQ score difference 0.57, 95% CI −0.64 to 1.78; *p* = 0.354) or secondary patient outcomes. The RMDQ difference met the predefined criterion to indicate noninferiority. One control group participant experienced an activity-related gluteal tear, with no other adverse events recorded. Intervention group GPs had improvements in attitudes, knowledge, and confidence compared with control group GPs. Intervention group GP LBP management behaviour became more guideline concordant than the control group. In cost-effectiveness, the intervention dominated control with lower costs and higher Quality-Adjusted Life Year (QALY) gains. Limitations of this study were that although adequately powered for primary outcome assessment, the study was not powered for evaluating some employment, healthcare use, and economic outcomes. It was also not possible for research nurses (responsible for patient recruitment) to be masked on group allocation for practices.

**Conclusions:**

Findings from this study suggest that the FREE approach improves GP concordance with LBP guideline recommendations but does not improve patient recovery outcomes compared with usual care. The FREE approach may reduce unnecessary healthcare use and produce economic benefits. Work participation or health resource use should be considered for primary outcome assessment in future trials of undifferentiated LBP.

**Trial registration:**

ACTRN12616000888460

## Abstract 2

Ben Darlow and colleagues present results of their trial to aid GPs identify lower pack pain earlier using a new approach.

## Author summary

**Why was this study done?:**

- Low back pain (LBP) is the world’s leading cause of disability and a common reason to consult a general practitioner (GP).
- There is broad international consensus on the best ways to manage LBP, but many of these recommendations have not been integrated into standard primary care clinical practice.
- This gap between evidence and practice results in worse recovery outcomes and overdiagnosis, overtreatment, and exposure to unnecessary risk of harm for people with LBP.

**What did the researchers do and find?:**

- We developed the Fear Reduction Exercised Early (FREE) approach to LBP for use in routine GP clinical consultations to bridge the evidence–practice gap.
- We randomly allocated groups of GPs to either continue with usual clinical care or receive training in the FREE approach in conjunction with support to integrate the approach into their clinical practice. We measured outcomes for GPs and patients with LBP who consulted these GPs.
- We found that GPs who received the FREE training had changes in their attitudes, knowledge, and confidence, and their clinical practice became more aligned with best-practice guidelines.
- We found that recovery (disability and pain) was similar for patients who consulted intervention or control group GPs, but those who consulted a GP trained in the FREE approach used less additional healthcare and had lower costs.

**What do these findings mean?:**

- These findings challenge the assumption that improving GP concordance with guidelines for LBP will improve patient recovery outcomes.
- These findings suggest that FREE may be an effective way to improve GP contributions to LBP care and improve efficiency without negatively impacting on patient recovery.
- Extending the intervention to other parts of the system, such as employers and other healthcare providers, may provide an opportunity to optimise the impact of FREE through reinforcing key messages and enabling recommended patient behaviours.
- Future studies should consider using participation measures, such as days off work, or healthcare resource use, as primary outcomes to assess the impact of intervention on the burden of LBP.

## Introduction

Low back pain (LBP) is a highly prevalent, complex, and expensive health condition and has been the world’s leading cause of disability since 1990 [1,2]. Globally, a substantial gap exists between current evidence on treating back pain and actual practice [3,4]. This gap results in overuse of opioid medication, spinal imaging, interventional procedures (such as guided injections), and surgery [3]. Use of these interventions when not indicated provides poor value to both patients and health systems through overdiagnosis, overtreatment, and exposure to unnecessary risk of harm [3,5].

There is broad international consensus on LBP best-practice recommendations [3]. It is assumed that implementation of these recommendations will improve healthcare outcomes and potentially reduce costs by preventing use of harmful and wasteful management approaches [3,5,6]. However, few studies have examined the impact of general practice implementation of LBP best-practice recommendations, and few of these have demonstrated improvements in patient outcomes or reduction in healthcare costs [7,8].

The Fear Reduction Exercised Early (FREE) approach is a multifaceted primary care intervention developed in New Zealand for general practitioner (GP) use in routine clinical consultation. The FREE approach aligns with LBP guidelines by empowering primary care management of LBP, enabling GPs to provide evidence-based education and advice, encouraging activity and work participation, integrating a biopsychosocial approach, and discouraging interventions with low beneficial value [2,3]. The FREE approach also adheres to recommended strategies to overcome implementation barriers by including GP education and training, electronic consultation support, and social interaction [9]. Pilot testing of the FREE approach found that GPs considered it to be acceptable and useful [10]. We aimed to determine (i) whether patients with LBP who receive care from GPs trained in the FREE approach have better outcomes than those who receive usual GP care; (ii) whether training GPs in the FREE approach influences their attitudes, knowledge, confidence, and clinical behaviour related to LBP; and (iii) whether training GPs in the FREE approach reduces economic costs associated with LBP.

## Methods

The trial was prospectively registered with the Australia New Zealand Clinical Trial Registry (ACTRN12616000888460) and is reported as per the Consolidated Standards Of Reporting Trials (CONSORT) guideline (S1 Checklist).

### Study design

We conducted a pragmatic cluster-randomised superiority trial in 8 general practices, with assessment of outcomes blinded to group allocation. In parallel, we also conducted a process evaluation to assess fidelity of intervention delivery by the GPs and a health economic evaluation. Cluster randomisation of general practices (rather than practitioners) was necessary to reduce risk of contamination associated with 1 GP providing 2 types of care or GPs discussing the intervention with colleagues in their practice.

### Participants

General practices with more than 3 full-time equivalent (FTE) GPs were recruited from the Hutt Valley region of New Zealand. All GPs working at these practices were invited to participate, with no further exclusion criteria.

All patients with LBP presenting to participating practices were screened for eligibility by a research nurse prior to their GP consultation. Patients were eligible if they were over 18 years of age and had LBP of any duration (with or without leg pain) as their main reason for consultation. Patients were excluded if they had received back surgery in the previous 6 months, had been unable to do their normal work for more than 3 of the last 6 months, had back pain due to a non–back-related condition (e.g., hip arthritis) or a serious health condition (e.g., cauda equina syndrome, spinal infection), had a concomitant health condition that meant they were unsuitable for trial participation (e.g., pregnancy or major psychological disturbance), or were unable to read or write in English.

Eligibility criteria changed early during the data collection phase (updated in the trial registration and presented in the published protocol) [11]. The protocol originally stated that the patient group would be limited to those who had experienced LBP for less than 6 weeks, were between 18 and 65 years, and had not seen a health professional about back pain in the last 3 months. These criteria were removed as these unnecessarily restricted patient eligibility and risked reducing the generalisability of findings.

All GP and patient participants gave written informed consent with optional additional consent (required from both GP and patient) to audio-record consultations. The trial was approved by the New Zealand Central Region Health and Disability Ethics Committee (16/CEN/43) and conducted in accordance with the published protocol [11].

### Randomisation and masking

General practices and GPs working in those practices were recruited prior to practice allocation to group. General practices were randomly assigned with a 1:1 allocation to either the intervention or control arm (i.e., all GPs in a practice were randomised to the same arm), using a computer-generated randomisation schedule stratified by the number of FTE GPs within the practice (≤8 versus >8 FTE). An independent statistician at a central administration site conducted the randomisation to ensure allocation concealment. Practice allocation was initially communicated only to the principal investigator so that intervention training and data collection dates could be arranged with practices. Practices and GPs were necessarily unblinded post randomisation to facilitate planning and delivery of intervention workshops and related data collection.

Patient participants were recruited post randomisation. All GP and patient data collection occurred post randomisation. To minimise the time between completing baseline measures and starting patient recruitment, baseline measures for GPs were completed by intervention arm GPs immediately prior to training delivery and by control arm GPs at 4 weeks prior to patient recruitment. GPs were aware of group allocation (but not intervention content) when they completed their baseline measures. Recruited patients were unaware of the trial’s existence and goals prior to presenting at their practice and were unaware that 2 different treatment approaches were being compared, meaning that they were masked to cluster allocation. Intervention and control consultations contained similar elements (history, examination, management) from a patient perspective. Research nurses at all practices used identical scripts, information sheets, and screening criteria to minimise potential recruitment bias. Data were cleaned and analysed by the trial statistician who was blinded to group allocation.

### Procedures

**Fig 1:**
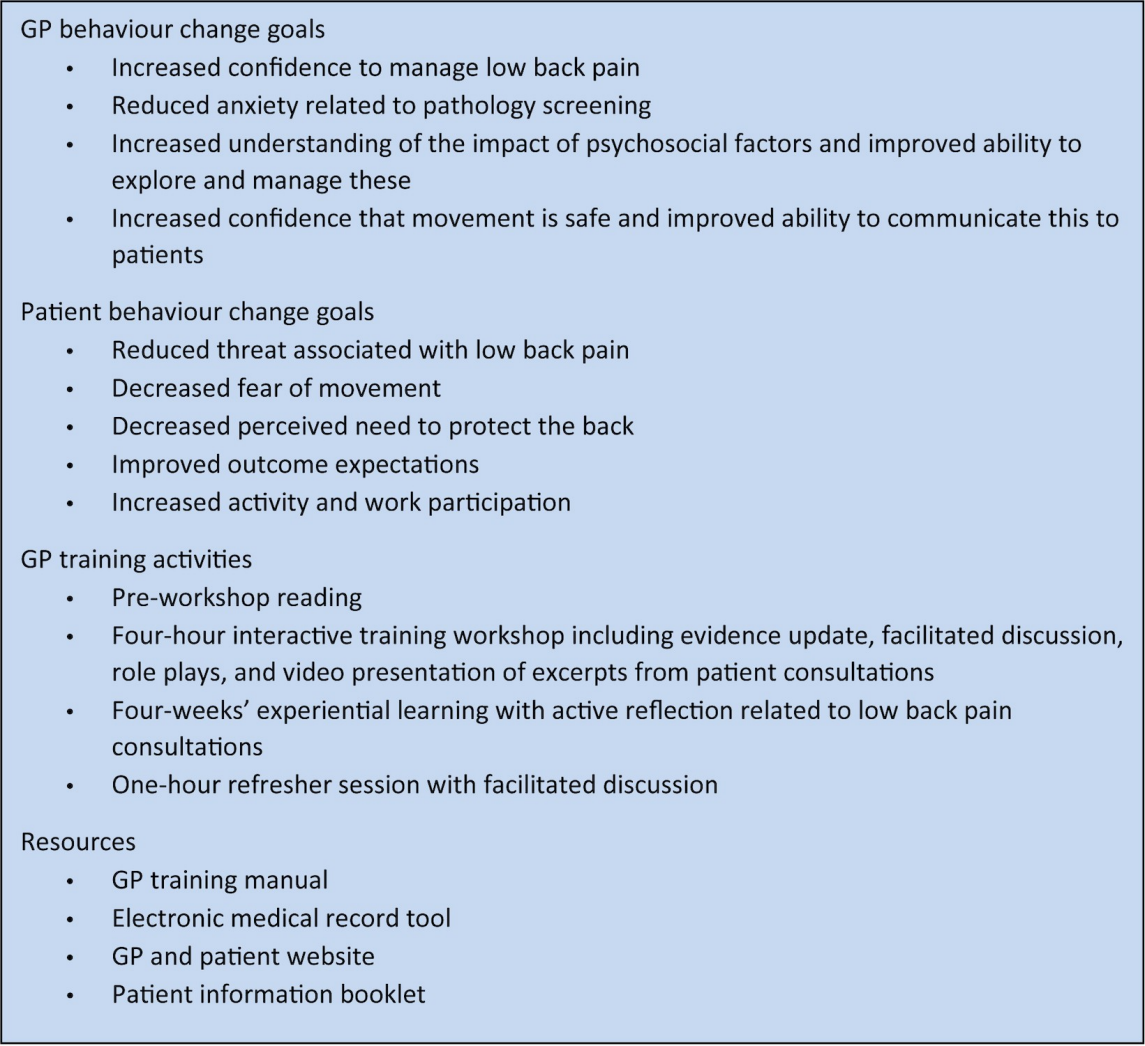
Overview of the FREE approach to LBP. FREE, Fear Reduction Exercised Early; GP, general practitioner; LBP, low back pain.

General practices in the control arm continued to provide usual care and did not receive specific training for this study (see S2 Checklist). GPs in the intervention arm were trained in the FREE approach to LBP (described previously [11]; see S3 Checklist). The FREE approach is delivered within standard GP consultations. It restructures and/or enhances the initial GP consultation for each LBP presentation and provides a framework and resources for approaching this and any subsequent consultations. FREE aims to optimise GP and patient behaviours and shift GP focus to addressing factors that can negatively influence outcomes. GPs were trained in the FREE approach through an initial 4-hour facilitated workshop, followed by a 4-week experiential learning period, and then a 1-hour refresher session. Intervention aims, activities, and resources are summarised in Fig 1. GPs were paid NZ$800 (GB£ 412.59) for time associated with training and NZ$20 (GB£ 10.32) for each of up to 3 patients with whom they used the FREE approach during the experiential learning period. Patient recruitment commenced after the experiential learning period in intervention practices had ended and at an equivalent interval of 4 weeks after completion of GP baseline measures in control practices. GPs in the intervention arm were asked to use the FREE approach to inform their consultations with patients who had LBP, although there was no compulsion or performance feedback, reflecting the pragmatic nature of the trial.

We collected GP data at baseline, 4 weeks (following the refresher session for intervention arm participants), and 4 months (timed to coincide with the anticipated end of patient recruitment). Patient data were collected at baseline (pre- and postconsultation for LBP), 2 weeks, 6 weeks, 3 months, and 6 months. Data were collected through surveys (electronic or postal), audio recording of consultations, and research nurse audit of clinical notes. Nonresponders were followed-up by telephone and text message.

### Outcomes

The primary patient-level outcome was disability at 6 months, measured with the Roland Morris Disability Questionnaire (RMDQ; scale 0–24; higher scores indicate greater disability) [12]. Secondary patient outcomes were the Numeric Pain Rating Scale (NPRS), Numeric Disability Rating Scale (DRS), satisfaction, and health-related quality of life (EuroQoL-5D [EQ-5D]) [13–15]. These patient-reported outcomes were collected at all patient follow-up points. Psychosocial indices (fear avoidance, pain self-efficacy, catastrophisation, anxiety, expectation) were collected at baseline, 2 weeks, and 6 weeks (the normal recovery period for most LBP) to examine mechanisms by which FREE might impact on patient outcomes (S1 Appendix p 13). Health economic data and some secondary patient outcomes (number of days off work/on restricted duties and quantities of medications taken) were self-reported by patients through the Otago Costs and Consequences Questionnaire for Low Back Pain (OCC-Q-LBP) [16] at 2 weeks, 3 months, and 6 months.

There was active and passive surveillance of potential harms (S1 Appendix p 15). Patients and GPs reported all unexpected or serious health events on a continuous basis during the study and through each follow-up survey. Each report was initially investigated by blinded members of the research team contacting patients and GPs to explore severity and potential links to back pain or study involvement. Each Adverse Event Reporting Form was reviewed by an independent academic GP who was a member of the data monitoring committee and approved by the entire data monitoring committee.

GP self-reported outcomes were attitudes about LBP and impairment (Health Care Providers Pain and Impairment Relationship Scale [HC-PAIRS] [17]), GP confidence to manage LBP [18], back pain knowledge (Back Pain Attitudes Questionnaire [Back-PAQ] [19]), and recommendations about activity, work, and rest as reported by GPs for a LBP case vignette [20]. Observed GP behaviour outcomes were consultation content (coded by research nurses from electronic consultation notes using a structured template) and patient report of GP recommendations (collected from the patients in their postconsultation data collection questionnaire).

Treatment fidelity was analysed by a researcher blinded to group allocation (S1 Appendix p 24). One audio recording was randomly selected for each GP who had consented to audio recording and treated at least 1 patient participant who had also consented to recording. Recordings were analysed using a structured checklist containing multiple consultation behaviour items. Consultations that included at least 4 out of 5 integral elements of FREE were defined a priori as FREE concordant.

Study data were collected and managed using Research Electronic Data Capture (REDCap) tools hosted at the University of Otago [21].

### Statistical analysis

The study was designed [11] with 80% power (two-tailed α of 0.05) to detect a between-group difference at 6 months of 2.5 RMDQ points (minimal clinically important difference) [22] assuming SD = 6.0 [23]. This gave *n* = 91 participants per arm under an individually randomised trial; clustering by GPs inflated this to *n* = 110 participants from 22 GPs per arm (total *n* = 220 patients and 44 GPs; intraclass correlation coefficient (ICC) at a conservative 0.05, assuming 5 completing patients per GP). Assuming 80% participant retention gave a recruitment target of 275 patients; assuming 25% of GPs would not recruit any participants gave a target of 30 GPs recruited per arm (*n* = 60 total).

We conducted an intention-to-treat analysis in line with the statistical analysis plan included in the published protocol [11]. All patients were analysed in the group to which their participating GP was allocated. Clustering of responses for GP outcomes was handled by including a random effect for GP practice in analytical models (random intercept + slope for GP practice) and for patient outcomes by specifying random effects for the GP (random intercept + slope for the GP, whereas randomisation was at the practice level, clustering effects on outcome values were expected to be mostly driven by interpractitioner variability). The stratified randomisation was handled by including the practice size stratum identifier in analytical models (≤8 FTE GPs, >8 FTE GPs). Data were included for each follow-up instance (for patient outcomes: 2 weeks, 6 weeks, 3 months, and 6 months; not all outcomes were measured at all time points).

Continuous outcomes (e.g., RMDQ) were analysed using linear mixed models. Categorical outcomes (such as satisfaction levels) were compared using generalised linear mixed models (with ordered outcome variables treated as ordinal outcomes, using cumulative link mixed models). For patient outcomes, these models adjusted for baseline level of the outcome being measured (e.g., baseline RMDQ included as a covariate when looking at RMDQ during follow-up) along with important baseline covariates (age, gender, socioeconomic status, current back pain duration and nature [constant or episodic], receipt of recent or ongoing non-GP healthcare for back pain, previous history of back pain, baseline disability, and baseline psychological factors [pain self-efficacy and recovery expectations]) [24]. Differences in outcomes by study arm were modelled over time by including follow-up time as a model term, and using interaction terms for time crossed with intervention arm to assess differences in intervention effects at specific follow-up time points. Analysis of patient outcomes also adjusted for GP-level baseline HC-PAIRS scores as a measure of baseline treatment competence.

The published protocol specified that the intervention could be considered noninferior to current management if the lower bound of the 95% CI for the RMDQ mean difference sat above −2.5 points (the minimal clinically important difference [MCID] [22]) [11,25].

The protocol specified that missing data would be handled through use of linear mixed models, which assume that incomplete postbaseline outcome measures are missing at random (MAR) conditional on group and adjustment variables and any completed postbaseline measures [26]. However, a total of 14 participants did not have time to complete all psychosocial items prior to GP consultation. In order to include these participants, their baseline scores on these covariates were imputed using mean imputation to allow models to be adjusted for all planned covariates [27]. Because the original protocol did not specify any particular outcome for missing baseline covariates, sensitivity analyses (S1 Appendix p 7) report on outcomes when (a) these participants with missing baseline data were excluded from analysis and (b) those adjustment items with missing data were excluded from the analytical model. All patient and GP analyses were conducted using R version 3.4.1 (R Institute, Vienna, Austria) with mixed models using the nlme package for continuous outcomes and the ordinal package for ordered categorical outcomes. The ICC for clustering on the primary outcome (RMDQ difference at 6 months) was estimated in a simplified linear mixed model with no adjustment for confounders (see S1 Appendix p 4 for methods and ICCs for other outcomes).

Cost-utility analysis estimated mean incremental cost per quality-adjusted life year (QALY) and monetary incremental net benefit (INB) gained from healthcare system and societal perspectives and from the Accident Compensation Corporation (ACC; universal no fault insurance cover for all personal injury in New Zealand) perspective, at willingness-to-pay (WTP) thresholds of 1, 2, and 3× gross domestic product (GDP) per capita (2017 NZ$). Reference costs were assigned for (i) all healthcare items, to allow direct comparison and decrease patient recall requirements; (ii) paid work (based on gender- and age-specific mean income); and (iii) unpaid and/or voluntary work (based on the minimum wage).

Multiple imputation was used to address missing data in the cost and health utility outcome measures. Patterns of missing data and the correlations between variables were examined to determine appropriate imputation models for each variable [28]. Imputations were created using predictive mean matching (continuous variables) and logistic regression models (dichotomous variables). As cost data are typically highly skewed, a large number of imputed datasets (*m* = 50) were created to ensure cost estimates would not be driven solely by a small number of large imputed values in 1 treatment arm. All multiple imputation analyses were conducted using mice (version 2.46.0) in R version 3.5.0.

Comparison of days off work and days on restricted duties was conducted using the multiply-imputed versions of these 2 variables because these measures were accumulated over the entire follow-up period (and hence mixed models could not account for missing data because there were no interim data). Adjusted linear mixed models (allowing for clustering and adjusted for standard factors as above) were used to analyse these 2 outcomes for each of the 50 imputed data sets. The resulting estimates were recombined following the standard rules for pooling results across imputed data sets [29].

An independent data monitoring committee oversaw conduct of the trial. Data are deposited in the Dryad repository (https://datadryad.org/review?doi=doi:10.5061/dryad.4t375b2) [30].

## Results

**Fig 2:**
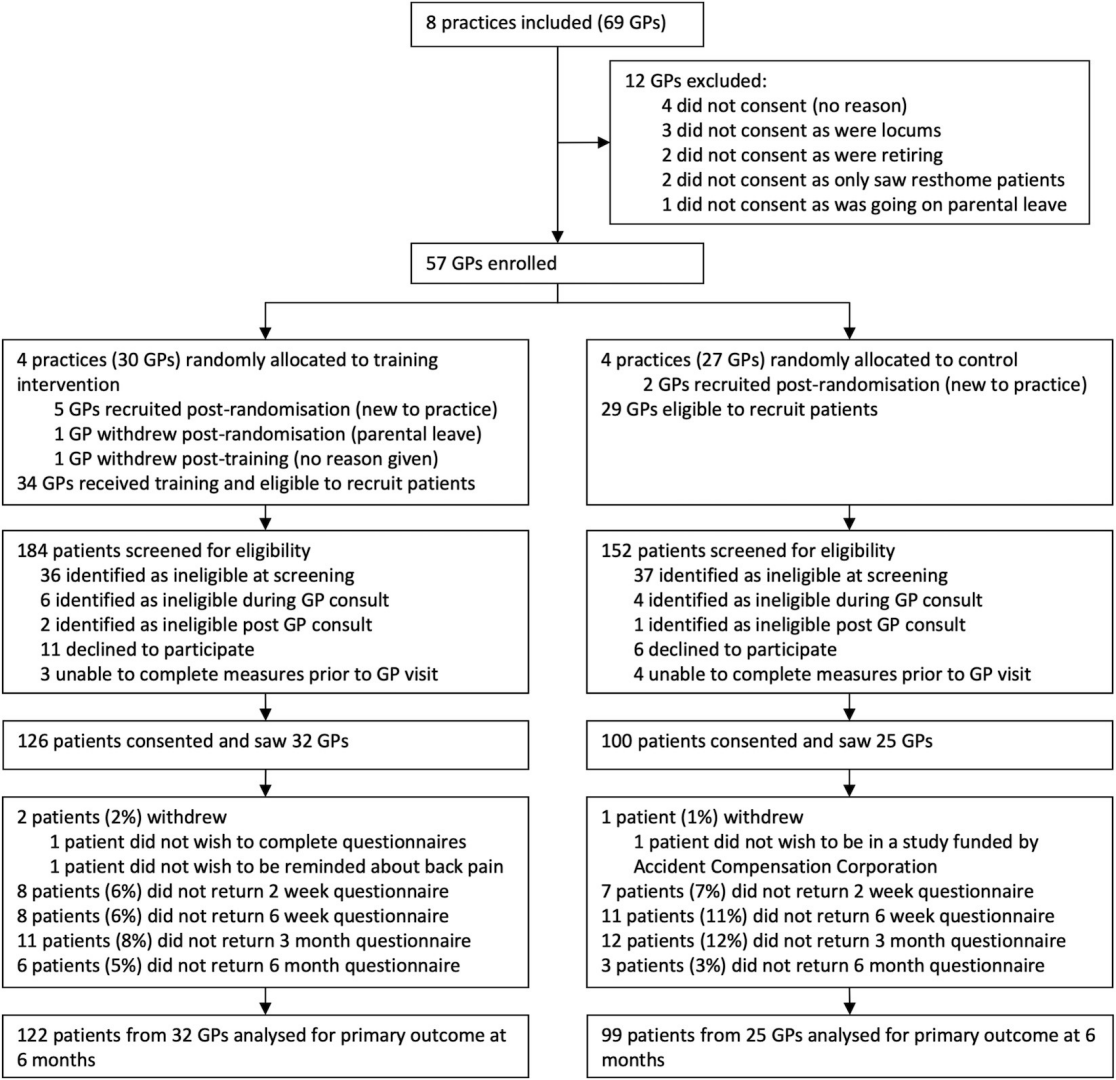
Trial profile. Supplementary data available in S1 Appendix S4 (p 3). GP, general practitioner.

Fig 2 shows the trial profile. Between 6 July and 8 August 2016, 57 GP participants were enrolled and subsequently randomised in practice clusters. Full data collection dates are in the S1 Appendix (p 2). An additional 7 GPs (5 intervention, 2 control) were recruited after joining practices post randomisation (but prior to their practice’s study participation), and 1 GP who was on parental leave at the time of her practice’s study participation withdrew prior to recruiting any participants. One intervention group GP withdrew post-training (baseline GP data only), but full data were collected for all other GP participants. GP characteristics were reasonably balanced between groups (Table 1).

**Table 1:** Baseline characteristics of GP participants.

GP characteristic	FREE (*n* = 34 GPs in 4 practices) Frequency (%) or mean (SD)	Control (*n* = 29 GPs in 4 practices) Frequency (%) or mean (SD)
Age (years)	47.0 (13.1)	45.9 (12.3)
Gender (female)	16 (47.1%)	15 (51.7%)
Years’ GP experience	14.4 (11.5)	14.7 (12.9)
Ethnicity^**a**^		
European	27 (81.8%)	18 (64.3%)
Māori^b^	1 (3.0%)	0 (0.0%)
Pacific	0 (0.0%)	1 (3.6%)
Asian	5 (15·2%)	8 (28.6%)
Other^c^	0 (0.0%)	1 (3.6%)
Previous back pain		
Never	2 (5.9%)	4 (13.8%)
Yes	28 (82.4%)	20 (69.0%)
Current	4 (11.8%)	5 (17.2%)
HC-PAIRS^d^	37.9 (9.7)	39.4 (9.7)
Back-PAQ^e^	18.1 (15.3)	14.5 (12.7)
Confidence^f^	9.7 (2.7)	9.3 (3.6)
Vignette guideline concordance		
Activity	23 (67.6%)	18 (62.1%)
Work	24 (70.6%)	21 (72.4%)
Rest	30 (88.2%)	23 (79.3%)

^a^Participants could select more than one option.

^b^Māori are the indigenous people of New Zealand.

^c^Other includes people who define their ethnicity as Middle Eastern, Latin American, African.

^d^Range 13 to 91, higher scores represent stronger attitudes that pain justifies disability and activity limitation.

^e^Range -68 to 68, lower scores represent more negative (or unhelpful) beliefs.

^f^Confidence to manage LBP measured with Provider Self Confidence Tool, range 4 to 16, lower scores indicate more confidence.

**Abbreviations:** Back-PAQ, Back Pain Attitudes Questionnaire; FREE, Fear Reduction Exercised Early; GP, general practitioner; HC-PAIRS, Health Care Providers’ Pain And Impairment Relationship Scale

Between 23 September 2016 and 31 July 2017, 140 eligible patient participants presented to intervention practices (126 enrolled) and 110 eligible patient participants presented to control practices (100 enrolled). Sufficient data were available to include 98% of patient participants in primary outcome analysis. All patient follow-up data were collected by 6 March 2018. Patient participant characteristics were reasonably balanced between groups (Table 2), although there were slight differences in participant ethnicity profiles, and the FREE arm had a slightly higher deprivation profile than the control arm. Most patients had back pain for less than 6 weeks (88%), currently had leg pain (67%), and had experienced previous back pain (83%). Thirty percent had not previously sought healthcare for back pain.

**Table 2:** Baseline characteristics of patient participants.

Patient characteristic	FREE (*n* = 126, recruited by 32 GPs) Frequency (%) or mean (SD)	Control (*n* = 100, recruited by 25 GPs) Frequency (%) or mean (SD)
Age (years)	46.2 (14.5)	45.9 (14.4)
Gender (female)	60 (47.6%)	45 (45.0%)
Ethnicity^a^		
European	82 (65.1%)	77 (77.0%)
Māori^b^	18 (14.3%)	14 (14.0%)
Pacific	13 (10.3%)	5 (5.0%)
Asian	12 (9.5%)	3 (3.0%)
Other^c^	1 (0.8%)	1 (1.0%)
NZDep quintile^d^		
1	24 (19.0%)	24 (24.0%)
2	25 (19.8%)	12 (12.0%)
3	20 (15.9%)	33 (33.0%)
4	22 (17.5%)	19 (19.0%)
5	35 (27.8%)	12 (12.0%)
Back pain duration^e^		
0–1 week	89 (71.2%)	76 (76.0%)
2–5 weeks	19 (15.2%)	15 (15.0%)
6–11 weeks	7 (5.6%)	6 (6.0%)
3–5 months	7 (5.6%)	2 (2.0%)
≥6 months	3 (2.4%)	1 (1.0%)
Leg pain present	81 (66.4%)	62 (68.1%)
Previous back pain	106 (84.8%)	81 (81.0%)
Previous HCP care for back pain^f^		
In past	85 (67.5%)	64 (64.0%)
In past 3 months	10 (7.9%)	9 (9.0%)
Current	10 (7.9%)	8 (8.0%)
RMDQ	12.6 (5.4)	12.7 (5.3)
NPRS Back^g^	6.9 (1.9)	6.9 (1.7)
NPRS Leg^g^	3.5 (3.2)	3.6 (3.2)
DRS^g^	6.3 (2.3)	6.2 (2.1)
EQ-5D^g^	0.5 (0.2)	0.4 (0.2)
PSEQ-2^g^	4.0 (1.6)	4.2 (1.7)
Recovery expectations^g^		
4 weeks	5.0 (1.8)	5.1 (1.6)
3 months	5.5 (1.6)	5.5 (1.5)
Fear avoidance^g^	5.0 (1.5)	4.8 (1.6)
Anxiety^g^	4.7 (1.7)	4.5 (1.8)
Catastrophisation^g^	3.3 (2.0)	3.3 (2.0)

^a^participants could select more than one option.

^b^Māori are the indigenous people of New Zealand.

^c^Other includes people who define their ethnicity as, Middle Eastern, Latin American, African.

^d^Quintile 1 represents people living in the least deprived 20% of small areas, Quintile 5 represents people living in the most deprived 20% of small areas.

^e^Back pain duration is reported in number of complete weeks/months.

^f^Participants could select more than one option.

^g^Data missing at baseline are presented in the S1 Appendix p 7.

**Abbreviations:** DRS, disability rating scale; EQ-5D, EuroQoL-5D; FREE, Fear Reduction Exercised Early; GP, general practitioner; HCP, Health Care Professional; NPRS, Numeric Pain Rating Scale; NZDep, New Zealand index of socioeconomic deprivation; PSEQ-2, two item Pain Self-Efficacy Questionnaire; RMDQ, Roland Morris Disability Questionnaire

**Fig 3:**
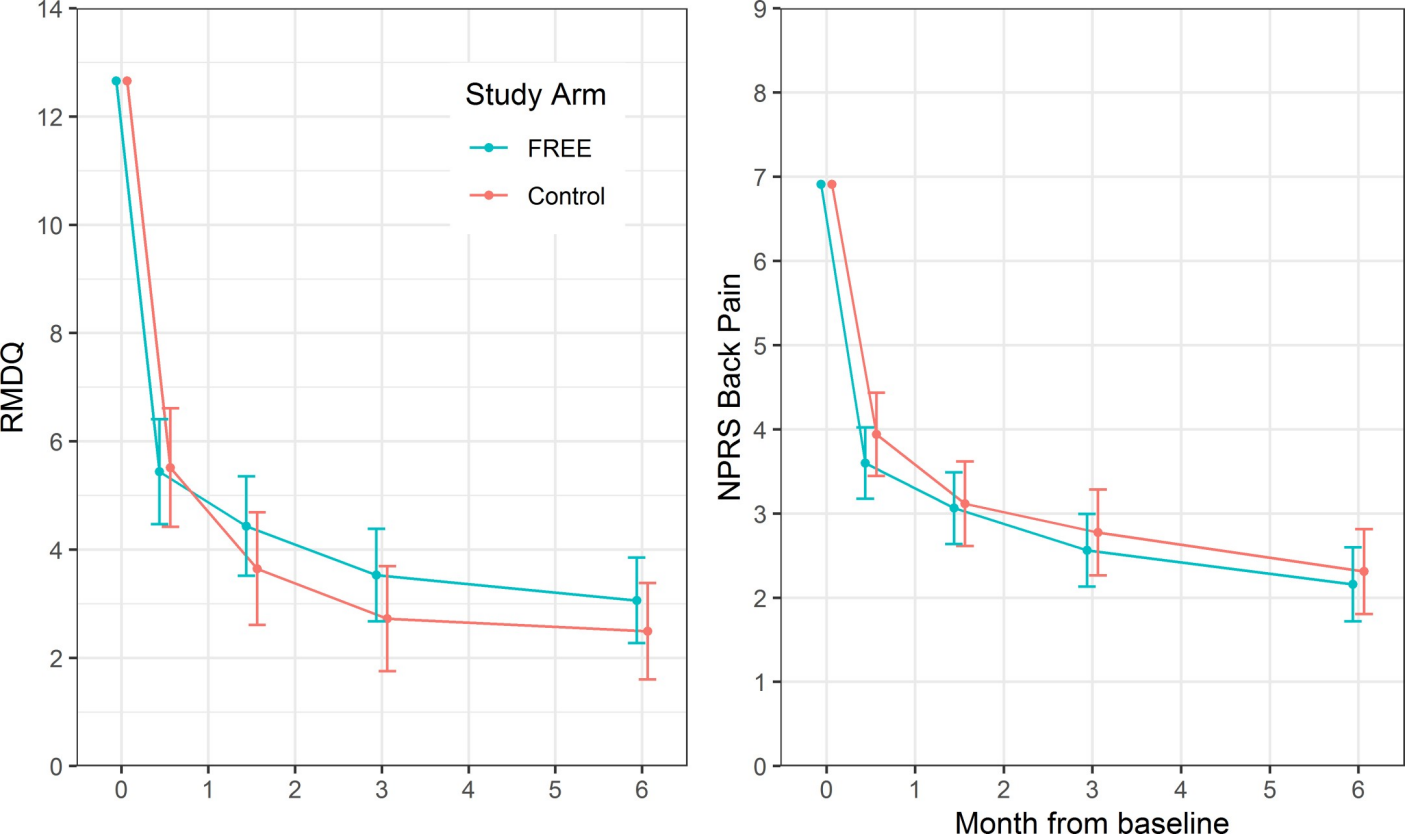
Mean patient participant RMDQ and NPRS scores by study arm at baseline and follow-up. Error bars represent 95% CIs of mean. FREE, Fear Reduction Exercised Early; NPRS, Numeric Pain Rating Scale; RMDQ, Roland Morris Disability Questionnaire.

Changes in patient outcomes are shown in Fig 3 and Table 3. The intervention did not reduce patient RMDQ scores compared with the control treatment at 6 months (mean difference 0·57, 95% CI −0·64 to 1·78; *p* = 0·354; higher scores in FREE arm) nor did it reduce NPRS back pain scores at 6 months (mean difference −0·15, −0·83 to 0·53; *p* = 0·664). The RMDQ difference met the predefined criterion to indicate noninferiority (lower end of CI for difference excluded MCID of 2.5 points relative to control). Sensitivity analyses examining the impact of FREE in patients meeting the original eligibility criteria and using different approaches to handle missing baseline covariate data returned highly comparable results (S1 Appendix p 5). The ICC (as a measure of clustering of outcomes by GP) for the RMDQ at 6 months was 0.16 (95% CI 0.09–0.24); ICCs for secondary outcomes are given in the S1 Appendix (p 4).

**Table 3:** Mean patient change scores at 2 weeks, 6 weeks, 3 months, and 6 months.

Characteristic	FREE mean change from baseline (95% CI)	Control mean change from baseline (95% CI)	Mean difference (95% CI)	*P* value
**RMDQ**^**1**^				
2 weeks	−7.22 (−8.19 to −6.25)	−7.14 (−8.24 to −6.05)	−0.08 (−1.56 to 1.41)	0.920
6 weeks	−8.22 (−9.14 to −7.31)	−9.01 (−10.05 to −7.97)	0.79 (−0.62 to 2.20)	0.271
3 months	−9.13 (−9.98 to −8.28)	−9.93 (−10.90 to −8.97)	0.80 (−0.51 to 2.12)	0.230
6 months	−9.60 (−10.39 to −8.81)	−10.17 (−11.06 to −9.28)	0.57 (−0.64 to 1.78)	0.354
**NPRS Back Pain**^**2**^				
2 weeks	−3.31 (−3.74 to −2.89)	−2.97 (−3.47 to −2.48)	−0.34 (−1.01 to 0.32)	0.316
6 weeks	−3.85 (−4.27 to −3.42)	−3.80 (−4.30 to −3.29)	−0.05 (−0.72 to 0.62)	0.878
3 months	−4.35 (−4.78 to −3.92)	−4.14 (−4.65 to −3.63)	−0.21 (−0.89 to 0.47)	0.544
6 months	−4.75 (−5.19 to −4.31)	−4.60 (−5.10 to −4.10)	−0.15 (−0.83 to 0.53)	0.664
**DRS**^**3**^				
2 weeks	−2.94 (−3.36 to −2.51)	−2.68 (−3.18 to −2.19)	−0.25 (−0.91 to 0.41)	0.456
6 weeks	−3.78 (−4.19 to −3.36)	−3.63 (−4.12 to −3.13)	−0.15 (−0.81 to 0.51)	0.660
3 months	−4.27 (−4.69 to −3.84)	−3.86 (−4.36 to −3.37)	−0.40 (−1.07 to 0.26)	0.235
6 months	−4.73 (−5.14 to −4.32)	−4.45 (−4.93 to −3.98)	−0.28 (−0.92 to 0.36)	0.394

^1^Number of participants contributing to regression models. Intervention: participants, *n* = 122; clusters, *n* = 32. Control: participants, *n* = 99; clusters, *n* = 25.

^2^Number of participants contributing to regression models. Intervention: participants, *n* = 119; clusters, *n* = 32. Control: participants, *n* = 90; clusters, *n* = 25.

^3^Number of participants contributing to regression models. Intervention: participants, *n* = 119; clusters, *n* = 32. Control: participants, *n* = 90; clusters, *n* = 25.

**Abbreviations:** DRS, disability rating scale; FREE, Fear Reduction Exercised Early; NPRS, Numeric Pain Rating Scale; RMDQ, Roland Morris Disability Questionnaire

GP participants did not report any adverse events or incidents of serious pathology during the trial. Patient participants reported 55 health events that they considered to be serious or unexpected, and these were investigated as potential adverse events. No serious adverse events were reported. A full description of all potential harms is presented in the S1 Appendix (p 15). In brief, of these 55 patient-reported events, 26 were classified as mild (17 intervention; 9 control), 24 were classified as moderate (14 intervention; 10 control), and 5 as serious (4 intervention; 1 control). Only 1 (moderate) health event was classified as potentially being related to study participation, and this affected a control group participant who experienced an activity-related gluteal tear during recovery from LBP. All serious health events reported were unrelated to study participation; however, 3 of these resulted in participants being excluded from the trial because these serious conditions (spinal infection, common iliac artery stenosis, and pulmonary embolism) were criteria for exclusion.

With respect to psychosocial indices, there was a significant reduction in patient fear avoidance at 2 weeks in the FREE arm (mean difference −0.65, 95% CI −1.16 to −0.14; *p* = 0.013), but not at 6 weeks (mean difference −0.22, 95% CI −0.80 to 0.42; *p* = 0.42). No differences were seen in patient pain self-efficacy, recovery expectations, anxiety, or catastrophisation (S1 Appendix p 13).

Patients in the FREE arm were slightly less satisfied with the information received immediately following their consultation, but no other satisfaction differences were observed between groups (S1 Appendix p 10).

**Fig 4:**
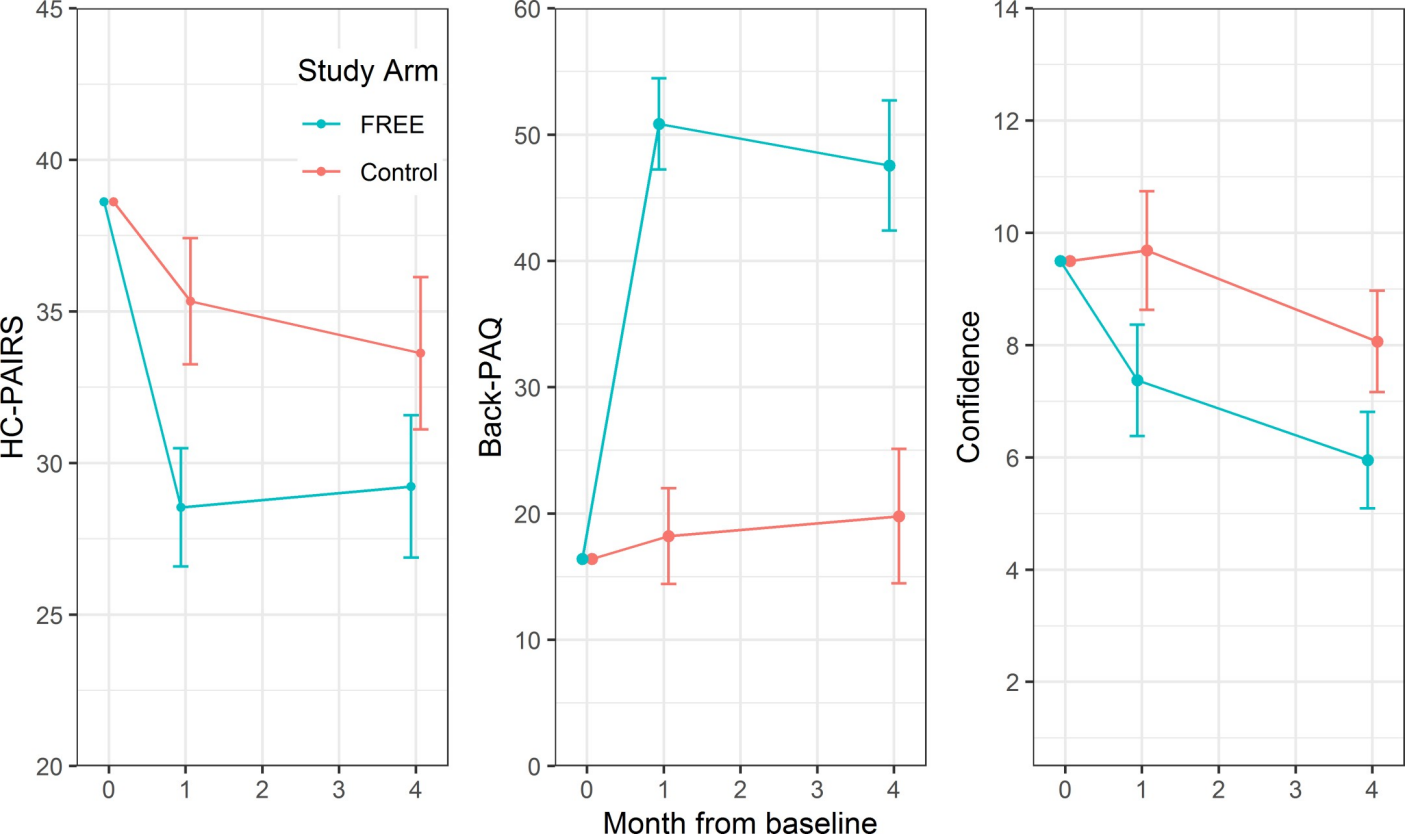
Mean GP participant HC-PAIRS, Back-PAQ, and confidence to manage LBP scores by study arm at baseline and follow-up. Error bars represent 95% CIs of mean. Back-PAQ, Back Pain Attitudes Questionnaire; FREE, Fear Reduction Exercised Early; GP, general practitioner; HC-PAIRS, Health Care Providers Pain and Impairment Relationship Scale.

Comprehensive GP results are presented in the S1 Appendix (p 18). Changes in GP attitude, knowledge, and confidence scores are shown in Fig 4 and Table 4. GPs trained in the FREE approach had significantly greater improvements in HC-PAIRS scores at 4 weeks (mean difference −6.80; 95% CI −9.62 to −3.98; *p* < 0.001) and at 4 months (−4.39, 95% CI −7.80 to −0.99; *p* = 0.011), Back-PAQ scores at 4 weeks (32.64, 95% CI 27.46 to 37.81; *p* = 0.002) and 4 months (27.76, 95% CI 20.45 to 35.08; *p* < 0.001), and confidence to manage LBP at 4 weeks (−2.31, 95% CI −3.75 to −0.88; *p* < 0.001) and 4 months (−2.11, 95% CI −3.35 to −0.88; *p* < 0.001).

**Table 4:** Mean GP change scores at 4 weeks and 4 months postintervention training.

Characteristics	FREE mean change from baseline (95% CI)	Control mean change from baseline (95% CI)	Mean difference (95% CI)	*P* value
**HC-PAIRS**^**1**^				
4 weeks	−10.08 (−12.02 to −8.13)	−3.28 (−5.36 to −1.20)	−6.80 (−9.62 to −3.98)	<0.001
4 months	−9.39 (−11.74 to −7.04)	−4.99 (−7.50 to −2.48)	−4.39 (−7.80 to −0.99)	0.011
**Back-PAQ**^**2**^				
4 weeks	34.44 (30.84 to 38.04)	1.81 (−1.98 to 5.59)	32.64 (27.46 to 37.81)	0.002
4 months	31.15 (26.01 to 36.29)	3.39 (−1.93 to 8.70)	27.76 (20.45 to 35.08)	<0.001
**Confidence**^**3**^				
4 weeks	−2.12 (−3.11 to −1.14)	0.19 (−0.87 to 1.24)	−2.31 (−3.75 to −0.88)	<0.001
4 months	−3.55 (−4.40 to −2.69)	−1.43 (−2.34 to −0.53)	−2.11 (−3.35 to −0.88)	<0.001

^1^Range 13 to 91, higher scores represent stronger attitudes that pain justifies disability and activity limitation. Intervention: GPs, *n* = 33; clusters, *n* = 4. Control: GPs, *n* = 29; clusters, *n* = 4.

^2^Range -68 to 68, lower scores represent more negative (or unhelpful) beliefs. Intervention: GPs, *n* = 33; clusters, *n* = 4. Control: GPs, *n* = 29; clusters, *n* = 4.

^3^Confidence to manage LBP measured with Provider Self Confidence Tool, range 4 to 16, lower scores indicate more confidence. Intervention: GPs, *n* = 33; clusters, *n* = 4. Control: GPs, *n* = 29; clusters, *n* = 4.

**Abbreviations:** Back-PAQ, Back Pain Attitudes Questionnaire; FREE, Fear Reduction Exercised Early; GP, general practitioner; HC-PAIRS, Health Care Providers’ Pain And Impairment Relationship Scale

Analysis of GP clinical notes and patient report post consultation found that intervention arm GPs delivered more guideline-consistent care than control-arm GPs (S1 Appendix p 21). Patients who saw intervention-arm GPs were more likely to report receiving advice to continue with normal activity (68.6%, 95% CI 60.8 to 75.5 versus 43.7%, 95% CI 32.9 to 55.0) and less likely to report being referred for physiotherapy, osteopathy, chiropractic, and/or acupuncture (24.6%, 95% CI 16.4 to 35.1 versus 68.0%, 95% CI 58.2 to 76.4). Blinded analysis of one randomly selected audio recording for each recruiting GP with available recordings found that 82.6% (95% CI 61.2 to 95.0) of intervention GPs met the predefined threshold for FREE concordance compared with 0% (95% CI 0.0 to 11.7) of control group GPs. High proportions of GPs in both arms reported guideline-consistent management advice in relation to the clinical vignette. GPs in the FREE arm were more likely to give guideline-consistent activity vignette recommendations at 4 months (unadjusted estimates: 87.9% versus 69.0% for FREE and control, respectively; odds ratio [OR] 4.6, 95% CI 0.7 to 30.5), but there were no differences in guideline consistency of work or rest vignette recommendations (S1 Appendix p 26).

**Fig 5:**
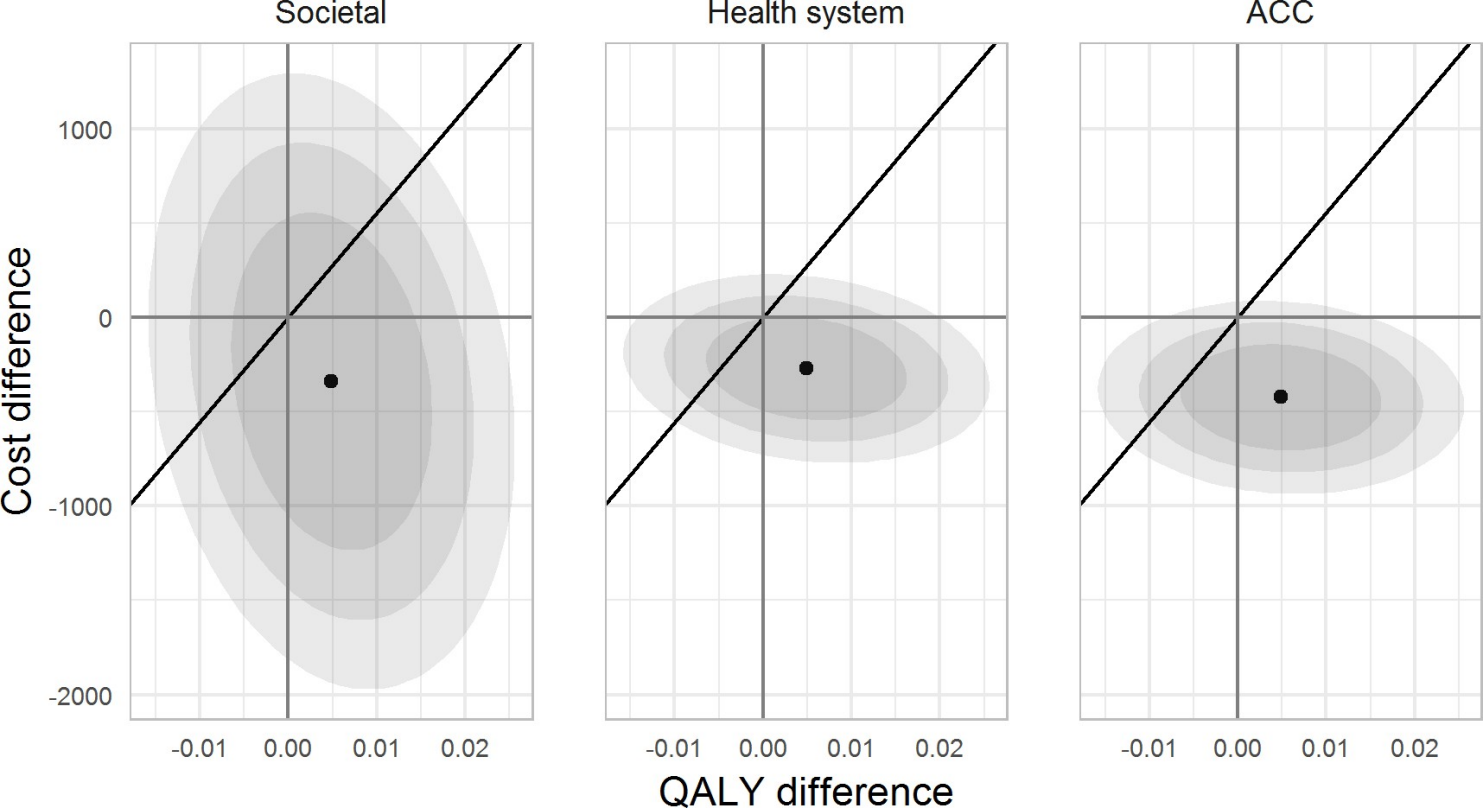
Cost-effectiveness plane. Shaded areas show 50% (darker), 75%, and 90% (lighter) confidence ellipses. Solid line shows WTP threshold at 1× GDP per capita; areas below and to the right of the line indicate the intervention is cost-effective relative to control; above and to the left indicate the intervention is not cost-effective. ACC, Accident Compensation Corporation; GDP, gross domestic product; QALY, quality-adjusted life year; WTP, willingness to pay.

Details of healthcare resource use, healthcare costs, and health-related quality of life (EQ-5D scores and QALYs) are provided in the S1 Appendix (p 29). The cost-effectiveness plane is shown in Fig 5. The FREE intervention dominated control, with higher QALY gains and lower costs, resulting in negative cost-utility ratios (−NZ$69,002; −$55,223; and −$86,440 per QALY gained from the societal, health system, and ACC perspectives, respectively). The FREE intervention demonstrated an INMB of $609 (95% CI −1,385 to 2,603), $542 (95% CI −678 to 1,762), and $695 (95% CI −496 to 1,885) from the societal, health system, and ACC perspectives, respectively, at the 1× GDP/capita WTP threshold (2017 NZ$55,615).

## Discussion

Findings from this trial suggest that patients with LBP who receive care from GPs trained in the FREE approach do not have improved disability or pain outcomes compared with those who receive usual care. In contrast, the FREE intervention improved GP attitudes, knowledge, and confidence and changed GP LBP management behaviours to be more guideline concordant. Economic evaluation was inconclusive but indicated the FREE approach may reduce the overall costs of LBP to the healthcare system and society.

Analyses using multiple consultation data sources demonstrated that the FREE intervention improved GP guideline concordance, countering systematic review findings that GP educational opportunities produce little or no improvement in GP guideline-consistent behaviour [31]. Notably, GPs trained in FREE were more likely to consider psychosocial factors, provide explanation, and provide advice to remain active. The FREE intervention appears to have improved GPs’ confidence in their ability to provide LBP care independently (i.e., without referral for additional healthcare); referral rates to rehabilitation providers were much lower in the intervention arm than in the control arm (which appeared high in comparison to international norms). Nonsignificant trends for reduced imaging and reduced specialist referral were also observed for intervention-arm GPs. The slight reduction in intervention-arm patient satisfaction is consistent with reduced satisfaction reported in another recent GP guideline implementation study [8]. Those authors hypothesized this may be due to patients’ unfulfilled expectations or disagreeing with guideline concordant advice [8]. Further research is required to explore patient responses to guideline concordant care.

Our finding that improving GP behaviours toward guideline concordance did not improve patient recovery outcomes challenges the assumption that improved guideline concordance will improve healthcare outcomes [3]. The FREE intervention has a large focus on education and addressing psychosocial barriers to recovery and prescribing simple exercises, because adding these guideline concordant interventions has been found to improve the cost-effectiveness of GP care of LBP [6]. FREE is delivered during a standard consultation, raising the possibility that patients did not receive sufficient education to improve outcomes. However, adding 2 hours of primary care–based intensive education to recommended first-line care for people with LBP at risk of poor outcomes did not improve pain and disability outcomes in a recent study [32].

Our findings suggest the FREE intervention achieved GP behaviour change targets with respect to exploring psychosocial barriers to recovery and providing reassurance and explanation in relation to these barriers but that these changes did not improve patient recovery outcomes. Jellema and colleagues [33] did not find improved patient outcomes after training GPs to identify and manage psychosocial factors, concluding that this was due to GPs neither adequately identifying these factors nor having sufficient time to address these. The Subgroups for Targeted Treatment (STarT) Back tool screens for psychosocial factors so that care can be matched to patient risk of poor recovery, including provision of psychosocially informed physiotherapy to those at high risk of poor recovery outcomes [23]. The STarT Back approach was found to produce a statistically significant but not clinically meaningful improvement in recovery outcomes in the United Kingdom [23], but the recent United States Matching Appropriate Treatments to Consumer Healthcare needs (MATCH) trial did not find improvements in clinical behaviour or patient outcomes [34]. Although psychosocial factors are associated with recovery outcomes, findings from these trials do not provide strong support for the theory that addressing psychosocial factors will improve recovery outcomes. Healthcare utilisation has been proposed as a measure of patient reassurance [35]. Our trial and the United Kingdom STarT Back trial both found reduced healthcare utilisation in the intervention arms. This suggests that exploring psychosocial factors and providing appropriate reassurance might reduce healthcare use while achieving similar recovery outcomes [23].

This trial had a number of strengths and limitations. Recruitment of patients into primary care randomised controlled trials can be challenging [36]. The current trial attempted to optimise recruitment by placing research nurses in practices to recruit patient participants as they presented to the practice and hence before consulting their GP. To our knowledge, this is the first study that has recruited people presenting with LBP prior to GP consultation. This process avoided GP recruitment bias and time delays between initial healthcare interaction and completion of baseline measures, as well as enabling patients to remain blind to the intervention. One limitation is that it was not possible to mask research nurses to the group allocation of the practices in which they were based. To mitigate this potential for recruitment bias, we used identical scripts, information sheets, and screening criteria. Mean outcome variable values (i.e., clinical characteristics) at baseline were almost identical in the 2 study arms, suggesting that there was no particular bias arising in the clinical characteristics of patients recruited in the 2 study arms. Recruitment prior to consultation also enabled exploration of the content of the consultations, collection of patient perspectives with reduced recall bias, and analysis of the impact of that consultation on outcome and process variables such as psychosocial indices. The collection of consult-related information from multiple sources (audio recording, direct patient report immediately post consult, electronic consultation note audit) was important to provide a comprehensive understanding of the consultation. Notwithstanding these benefits, the limited time between practice presentation and being called for their GP consult meant that some patient participants were not able to complete all baseline measures, and some imputation was required to enable the prespecified adjustment of primary outcome analysis. Sensitivity analyses indicated this did not affect the interpretation of results.

Excellent follow-up rates (97% intervention and 99% control contributing data to final analysis) enabled the estimated target number of participants to adequately power the primary outcome analysis despite not meeting recruitment targets. Cluster effects (ICCs) were greater than anticipated, but we also recruited from more clusters than anticipated (a higher number of smaller clusters is preferable). The precision of the primary outcome (reflected in its 95% CI) makes us confident that the FREE intervention did not produce meaningfully better (or worse) disability outcomes than usual care. Notwithstanding this, the study was underpowered for some employment, healthcare use, and economic outcomes.

Control group participants in this study demonstrated large improvements in disability. As the mean control group RMDQ score was 2.5 points at 6 months, it would not have been possible for the FREE arm to show a clinically meaningful improvement on the RMDQ measure. Although the RMDQ is recommended as a core LBP outcome measure [14], these results raise questions about its appropriateness as a primary outcome for LBP in this population. Future trials might consider participation measures, such as days off work, or healthcare resource use, as primary outcomes to assess these changes in the burden of back pain without being limited by floor effects observed in the long-term patient-reported outcome measures used in this study. This would, however, require substantially larger sample sizes than measures of patient-reported pain or disability.

A major strength for generalisability of findings is that this study was delivered in a real-life clinical context. Broad patient inclusion criteria were used to best match daily practice. Consultation content and intervention fidelity were measured, but GP behaviour was not controlled. These characteristics increase confidence that FREE could be implemented into standard practice and could be applied to undifferentiated back pain. A parallel implementation study (reported separately) enabled exploration of implementation constructs without gathering additional data from intervention-arm participants (such as qualitative interviews) that may have acted as co-interventions.

The FREE approach met the predefined noninferiority criterion for the RMDQ [11]. Trends towards reduced healthcare use, reduced time off work, and reduced overall costs in the intervention group were consistent with the measured differences in GP behaviour and the targets of FREE approach training, but estimates were not sufficiently precise to confirm cost savings. These findings suggest that FREE may be an effective mechanism to improve GP contributions to LBP care and improve efficiency without negatively impacting patient outcomes. Extending the intervention to other parts of the system, such as employers and other healthcare providers, may provide an opportunity to optimise the impact of FREE through reinforcing key messages and enabling recommended patient behaviours.

This trial demonstrated that a brief training intervention with supporting resources can improve GP attitudes, knowledge, confidence, and clinical behaviour related to LBP management. The intervention did not improve patient disability or pain outcomes more than usual care, but may reduce unnecessary healthcare use, thereby reducing exposure to harm and enabling improved allocation of healthcare resources.

## Supporting information

S1 Checklist CONSORT checklist. CONSORT, Consolidated Standards of Reporting Trials
(DOCX)

S2 Checklist Control description according to the TIDieR checklist. TIDieR, Template for Intervention Description and Replicaiton
(DOCX)

S3 Checklist Intervention description according to the TIDieR checklist. TIDieR, Template for Intervention Description and Replication
(DOCX)

S1 Appendix Additional reporting of methods, analysis, and results. (DOCX)

## Abbreviations

ACC: Accident Compensation Corporation
Back-PAQ: Back Pain Attitudes Questionnaire
CONSORT: Consolidated Standards Of Reporting Trials
DRS: Disability Rating Scale
EQ-5D: EuroQoL-5D
FREE: Fear Reduction Exercised Early
FTE: full-time equivalent
GDP: gross domestic product
GP: general practitioner
HC-PAIRS: Health Care Providers Pain and Impairment Relationship Scale
ICC: intraclass correlation coefficient
INB: Incremental Net Benefit
LBP: low back pain
MAR: missing at random
MCID: minimal clinically important difference
NPRS: Numeric Pain Rating Scale
NZDep: New Zealand index of socioeconomic deprivation
OCC-Q-LBP: Otago Costs and Consequences Questionnaire for Low Back Pain
OR: odds ratio
PSEQ-2: Two item Pain Self-Efficacy Questionnaire
QALY: Quality-Adjusted Life Year
REDCap: Research Electronic Data Capture
RMDQ: Roland Morris Disability Questionnaire
STarT: Subgroups for Targeted Treatment
MATCH: Matching Appropriate Treatments to Consumer Healthcare needs
WTP: willingness to pay
