# Supplementary material for: The Fear Reduction Exercised Early (FREE) approach to management of low back pain in general practice: A pragmatic cluster-randomised controlled trial
Source: PLoS Med. 2019 Sep 9;16(9):e1002897. doi: 10.1371/journal.pmed.1002897 (PMC6733445; doi:10.1371/journal.pmed.1002897)
Supplement: S2 Checklist — TIDieR, Template for Intervention Description and Replicaiton (DOCX) [file pmed.1002897.s002.docx]

**S2 Checklist. Control description according to the Template for Intervention Description and Replication (TIDieR) Checklist.**

**Darlow B, Stanley J, Dean S, Abbott JH, Garrett S, Wilson W, Mathieson F, Dowell A (2019) *The Fear Reduction Exercised Early (FREE) approach to management of low back pain in general practice: a pragmatic cluster-randomised controlled trial* published in PLOS Medicine**

| **Item number** | **Item** |
| --- | --- |
|  |  |
|  | **BRIEF NAME** |
| **1.** | Usual care |
|  | **WHY** |
| **2.** | This was the best care available locally outside of the trial at the time of the study. |
|  | **WHAT** |
| **3.** | No materials were actively provided to general practitioners but they had full access to current low back pain guidelines and management pathways. |
| **4.** | Procedures:  General practitioners did not receive specific training for this study.  Patients received usual general practice care for low back pain. |
|  | **WHO PROVIDED** |
| **5.** | The patient participants were treated by general practitioners. All general practitioner participants were registered medical practitioners working in a general practice and had a mean 14·7 (SD 12.9) years’ general practice experience. The general practitioners delivered the care as part of their usual clinical role and did not receive any additional payment for providing this. |
|  | **HOW** |
| **6.** | General practitioner participants did not receive specific training for this study.  Patient participants received individual care as per normal from their general practitioner. This always included a face-to-face consultation, but may have also included telephone follow-up or asynchronous email or web-based interactions. |
|  | **WHERE** |
| **7.** | Patient participants received care within their usual general practice. |
|  | **WHEN and HOW MUCH** |
| **8.** | Patient participants received care as determined by their general practitioner (and the patient’s own autonomy). This was not influenced by the study, but was monitored and reported. Mean consultation duration based on audio-recorded consultations (recruitment consultation) in the control arm was 16·5 minutes (SD = 4·1; 14·7 to 18·2), reflecting usual GP low back pain consultation lengths. Participants attended a mean of 1·0 additional GP consultation related to low back pain during the 6 month follow-up period (95% CI 0·5 to 1·5). |
|  | **TAILORING** |
| **9.** | General practitioners personalised care to each patient participant as per usual practice. |
|  | **MODIFICATIONS** |
| **10.^ǂ^** | The control condition was not modified during the course of the study. |
|  | **HOW WELL** |
| **11.** | Planned:  Control practice of general practitioners was assessed by:   - GP-recorded consultation content was coded by research nurses from electronic consultation notes using a structured template - Patient report of GP recommendations collected from the patients in their post-consultation data collection questionnaire - Audio-recording of consultations. One audio-recording was randomly selected for each GP who had consented to audio-recording and treated at least one patient participant who had also consented to recording. Recordings were analysed using a structured checklist containing multiple consultation behaviour items. |
| **12.^ǂ^** | Actual:  Analysis of GP clinical notes and patient report post-consultation found that control arm GPs delivered less guideline consistent care than interventions arm GPs. Patients who saw control arm GPs were less likely to report receiving advice to continue with normal activity (intervention - 68·6%, 60·8 to 75·5 vs. 43·7%, control - 32·9 to 55·0) and more likely to report being referred for physiotherapy/osteopathy/chiropractic/acupuncture (intervention - 24·6%, 16·4 to 35·1 vs. control - 68·0%, 58·2 to 76·4) (see supplementary appendix for more detail).  Blinded analysis of one randomly selected audio-recording for each recruiting GP with available recordings found that 82.6% (61·2 to 95·0) of intervention GPs met the predefined threshold for FREE concordance compared with 0% (0·0 to 11·7) of control group GPs (see supplementary appendix for more detail). |

**REFERENCE**

Hoffmann T, Glasziou P, Boutron I, Milne R, Perera R, Moher D, Altman D, Barbour V, Macdonald H, Johnston M, Lamb S, Dixon-Woods M, McCulloch P, Wyatt J, Chan A, Michie S. Better reporting of interventions: template for intervention description and replication (TIDieR) checklist and guide. BMJ. 2014;348:g1687.
