# Supplementary material for: The Fear Reduction Exercised Early (FREE) approach to management of low back pain in general practice: A pragmatic cluster-randomised controlled trial
Source: PLoS Med. 2019 Sep 9;16(9):e1002897. doi: 10.1371/journal.pmed.1002897 (PMC6733445; doi:10.1371/journal.pmed.1002897)
Supplement: S3 Checklist — TIDieR, Template for Intervention Description and Replication (DOCX) [file pmed.1002897.s003.docx]

**S3 Checklist. Intervention description according to the Template for Intervention Description and Replication (TIDieR) Checklist.**

**Darlow B, Stanley J, Dean S, Abbott JH, Garrett S, Wilson W, Mathieson F, Dowell A (2019) *The Fear Reduction Exercised Early (FREE) approach to management of low back pain in general practice: a pragmatic cluster-randomised controlled trial* published in PLOS Medicine**

| **S3. Intervention description according to the TIDieR (Template for Intervention Description and Replication) Checklist** | |
| --- | --- |
| **Item number** | **Item** |
|  |  |
|  | **BRIEF NAME** |
| **1.** | FREE (Fear Reduction Exercised Early) approach to low back pain. |
|  | **WHY** |
| **2.** | The FREE approach aligns with low back pain guidelines by empowering primary care management of low back pain, enabling general practitioners to provide evidence-based education and advice, encouraging activity and work participation, integrating a biopsychosocial approach, and discouraging interventions with low beneficial value.  General practitioner behaviour change goals:   - Increased confidence to manage low back pain - Reduced anxiety related to pathology screening - Increased understanding of the impact of psychosocial factors and improved ability to explore and manage these - Increased confidence that movement is safe and improved ability to communicate this to patients   Patient behaviour change goals:   - Reduced threat associated with low back pain - Decreased fear of movement - Decreased perceived need to protect the back - Improved outcome expectations - Increased activity and work participation   Full description of included behaviour change techniques is available with the published protocol (<https://trialsjournal.biomedcentral.com/articles/10.1186/s13063-017-2225-8>). |
|  | **WHAT** |
| **3.** | Materials:   - Pre-workshop reading - GP training manual - Electronic medical record tool - GP and patient website - Patient information booklet (provided to patient participants by GP participants)   Further information available in published protocol (<https://trialsjournal.biomedcentral.com/articles/10.1186/s13063-017-2225-8>).. |
| **4.** | Procedures:  General practitioners were trained in the FREE approach through an interactive training workshop that included an evidence update, facilitated discussion, role plays, and video presentation of excerpts from patient consultations. They then undertook four-weeks’ experiential learning with active reflection related to low back pain consultations, followed by participation in a refresher session with facilitated discussion.  General practitioners were asked to use the FREE approach to inform their consultations with patients who had LBP. There was no compulsion or performance feedback, reflecting the pragmatic nature of the effectiveness trial. The electronic medical record tool aided adherence to the suggested model of consultation and the patient information booklet supported key messages, however, there was no compulsion to use these resources. |
|  | **WHO PROVIDED** |
| **5.** | The general practitioner participants were trained by the first author (BD) who led the development of the intervention. He was a Musculoskeletal Physiotherapy Specialist with 18 years of clinical experience and an experienced educator at the time of the first training session. GPs were paid NZ$800 (£GBP 412.59) for time associated with training and NZ$20 (£GBP 10.32) for each of up to three patients with whom they used the FREE approach during the experiential learning period.  The patient participants were treated by general practitioners who were trained as described above. All general practitioner participants were registered medical practitioners working in a general practice and had a mean 14·4 (SD 11.5) years’ general practice experience. The general practitioners delivered the intervention as part of their usual clinical role and did not receive any additional payment for providing this. |
|  | **HOW** |
| **6.** | General practitioner participants were trained in practice groups through face-to-face workshops.  Patient participants received individual care as per normal from their general practitioner. This always included a face-to-face consultation, but may have also included telephone follow-up or asynchronous email or web-based interactions. |
|  | **WHERE** |
| **7.** | General practitioner training workshops took place within general practitioner participants’ general practices.  Patient participants received care within their usual general practice. |
|  | **WHEN and HOW MUCH** |
| **8.** | GPs participated in two training sessions; a four-hour initial workshop and a one-hour refresher session following four-weeks’ of experiential learning.  Patient participants received care as determined by their general practitioner (and the patient’s own autonomy). This was not controlled by the study, but was monitored and reported. Mean consultation duration based on audio-recorded consultations (recruitment consultation) in the intervention arm was 22·8 minutes (SD = 8·5; 95% CI 19·1 to 26·5). Participants attended a mean of 1·0 additional GP consultations related to low back pain during the 6 month follow-up period (95% CI 0·6 to 1·4). |
|  | **TAILORING** |
| **9.** | General practitioner training was delivered using standard pre-reading, presentation, training manual, role plays, and video excerpts from patient consultations. The facilitated discussion was adapted to each workshop depending on topics that arose.  General practitioners personalised the intervention to each patient participant. The FREE approach provides a framework to guide consultations, suggested areas of focus, and techniques and explanatory models to support key objectives but general practitioners were able to apply these (or not) as they wished within each consultation. |
|  | **MODIFICATIONS** |
| **10.^ǂ^** | The intervention was not modified during the course of the study. The electronic medical record tool was slightly adapted to improve usability following feedback from the first intervention practice. A new version of the electronic medical record tool was created to support the third intervention practice as they used a different software system, however, the tool functioned very similarly to that used in other practices. |
|  | **HOW WELL** |
| **11.** | Planned:  GP training workshop delivery was assessed using a workshop evaluation form.  Intervention fidelity of general practitioners was assessed by:   - GP-recorded consultation content was coded by research nurses from electronic consultation notes using a structured template - Patient report of GP recommendations collected from the patients in their post-consultation data collection questionnaire - Audio-recording of consultations. One audio-recording was randomly selected for each GP who had consented to audio-recording and treated at least one patient participant who had also consented to recording. Recordings were analysed using a structured checklist containing multiple consultation behaviour items.   No active strategies were used to maintain or improve intervention fidelity, consistent with the pragmatic nature of the trial.. |
| **12.^ǂ^** | Actual:  Workshop evaluations were completed by 31 GPs in the FREE arm. These GPs rated the initial training workshop highly (see supplementary appendix for more detail).  Analysis of GP clinical notes and patient report post-consultation found that intervention arm GPs delivered more guideline consistent care than control arm GPs. Patients who saw intervention arm GPs were more likely to report receiving advice to continue with normal activity (68·6%, 60·8 to 75·5 vs. 43·7%, 32·9 to 55·0) and less likely to report being referred for physiotherapy/osteopathy/chiropractic/acupuncture (24·6%, 16·4 to 35·1 vs. 68·0%, 58·2 to 76·4) (see supplementary appendix for more detail).  Blinded analysis of one randomly selected audio-recording for each recruiting GP with available recordings found that 82.6% (61·2 to 95·0) of intervention GPs met the predefined threshold for FREE concordance compared with 0% (0·0 to 11·7) of control group GPs (see supplementary appendix for more detail). |

**REFERENCE**

Hoffmann T, Glasziou P, Boutron I, Milne R, Perera R, Moher D, Altman D, Barbour V, Macdonald H, Johnston M, Lamb S, Dixon-Woods M, McCulloch P, Wyatt J, Chan A, Michie S. Better reporting of interventions: template for intervention description and replication (TIDieR) checklist and guide. BMJ. 2014;348:g1687.
