# Supplementary material for: The Fear Reduction Exercised Early (FREE) approach to management of low back pain in general practice: A pragmatic cluster-randomised controlled trial
Source: PLoS Med. 2019 Sep 9;16(9):e1002897. doi: 10.1371/journal.pmed.1002897 (PMC6733445; doi:10.1371/journal.pmed.1002897)
Supplement: S1 Appendix — (DOCX) [file pmed.1002897.s004.docx]

**S1 appendix: Additional reporting of methods, analysis and results.**

**Darlow B, Stanley J, Dean S, Abbott JH, Garrett S, Wilson W, Mathieson F, Dowell A (2019) *The Fear Reduction Exercised Early (FREE) approach to management of low back pain in general practice: a pragmatic cluster-randomised controlled trial* published in PLOS Medicine**

This appendix has been provided by the authors to give readers additional information about their trial.

**Contents**

[Participant data collection dates 2](#_Toc12459314)

[Supplement to participant flow diagram 3](#_Toc12459315)

[Patient outcomes 4](#_Toc12459316)

[Intra-class correlation coefficients for patient outcomes 4](#_Toc12459317)

[Change in patient eligibility criteria primary outcome sensitivity analysis 5](#_Toc12459318)

[Sensitivity analysis for primary outcome: participants missing baseline co-variates 7](#_Toc12459319)

[Number Needed to Treat Primary Outcome Analysis 9](#_Toc12459320)

[Patient satisfaction 10](#_Toc12459321)

[Psychosocial process measures 13](#_Toc12459322)

[Harms 15](#_Toc12459323)

[GP outcomes 18](#_Toc12459324)

[GP satisfaction with training 18](#_Toc12459325)

[Patient report of GP management recommendations 19](#_Toc12459326)

[Electronic consultation record audit 21](#_Toc12459327)

[Consultation audio recording and treatment fidelity assessment 24](#_Toc12459328)

[GP reported clinical management of vignette case 26](#_Toc12459329)

[Economic analyses 29](#_Toc12459330)

[Healthcare utilisation 29](#_Toc12459331)

[Cost-effectiveness evaluation 31](#_Toc12459332)

[Sensitivity analyses 33](#_Toc12459333)

[References 35](#_Toc12459334)

## Participant data collection dates

| ***Table S1*. Participant data collection dates for each practice cluster** | | | | | | | |
| --- | --- | --- | --- | --- | --- | --- | --- |
| **Group** | **Practice** | **GP clusters** | **GP baseline** | **GP 4 month** | **First patient recruited** | **Last patient recruited** | **Last patient follow-up** |
| Control | 1 | 5 | 23/08/2016 | 12/12/2016 | 29/9/2016 | 22/03/2017 | 20/09/2017 |
| Intervention | 2 | 5 | 25/08/2016 | 12/12/2016 | 25/10/2016 | 29/03/2017 | 27/09/2017 |
| Control | 3 | 10 | 5/09/2016 | 9/01/2017 | 13/10/2016 | 15/03/2017 | 13/09/2017 |
| Intervention | 4 | 12 | 7/09/2016 | 9/01/2017 | 21/10/2016 | 12/04/2017 | 27/09/2017 |
| Control | 5 | 9 | 16/01/2017 | 17/05/2017 | 28/02/2017 | 31/07/2017 | 29/01/2018 |
| Intervention | 6 | 9 | 18/01/2017 | 19/05/2017 | 6/03/2017 | 6/07/2017 | 4/01/2018 |
| Control | 7 | 5 | 27/01/2017 | 29/05/2017 | 7/03/2017 | 29/05/2017 | 27/11/2018 |
| Intervention | 8 | 8 | 7/02/2017 | 9/06/2017 | 27/03/2017 | 28/07/2017 | 26/01/2018 |

## Supplement to participant flow diagram

| ***Table S2*. Supplement to participant flow diagram** | | | | |
| --- | --- | --- | --- | --- |
|  | **FREE** | | **Control** | |
|  | n | Reason | n | Reason |
| **Identified as ineligible at screening** | **36** |  | **37** |  |
|  | 5 | Pain in wrong area | 9 | Pain in wrong area |
|  | 7 | Pain longer than 42 days* | 15 | Pain longer than 42 days* |
|  | 5 | Outside of the age range* | 5 | Outside of the age range* |
|  | 7 | Received care for low back pain in the last 3 months* | 3 | Received care for low back pain in the last 3 months* |
|  | 11 | Long-term back-related disability | 5 | Long-term back-related disability |
| **Identified as ineligible during GP consult** | **6** |  | **4** |  |
|  | 3 | Primary complaint not low back pain | 1 | Primary complaint not low back pain |
|  | 1 | Back pain due pneumonia | 1 | Back pain due to pelvic inflammatory disease or urinary tract infection |
|  | 1 | Back pain due to shingles | 1 | Back pain due to bodily distress syndrome |
|  | 1 | Concomitant major psychological disturbance that meant they were unsuitable for trial participation s | 1 | Long-term disabled (not reported at screening) |
| **Identified as ineligible post GP consult** | **2** |  | **1** |  |
|  | 1 | Back pain due to pulmonary embolism | 1 | Back pain due to spinal infection |
|  |  | Back pain due to common iliac artery stenosis (GP referred to vascular sugeon at time of initial consult) |  |  |
| **Withdrew** | **2** |  | **1** |  |
|  | 1 | Following baseline | 1 | Following baseline |
|  | 1 | Following 2 week survey |  |  |
| * These criteria were removed from 1 January 2017 as these unnecessarily restricted patient eligibility and risked reducing the generalisability of findings. | | | | |

## Patient outcomes

### Intra-class correlation coefficients for patient outcomes

#### Method

The intra-class correlation coefficient (ICC) is a measure of similarity of scores within clusters. ICCs were calculated for each patient reported outcome measure to estimate the impact of clustering by general practitioner (GP). Values of zero would indicate no clustering; values of one would indicate deterministic clustering (that is, all patients of any GP achieve the same outcome). Ninety-five per cent confidence intervals for the ICC were estimated through bootstrapping. The ICC was estimated using simplified linear mixed models with no adjustment for confounders, using the bootMer function in R’s lme4 package.^1^

#### Result

Table S3 presents the ICC estimates for each of the patient outcomes, with their 95% confidence intervals.

| ***Table S3*. Intra-class correlation coefficient (ICC, with 95% CI) for patient outcomes.** | |
| --- | --- |
| **Measure** | **ICC (95% CI)** |
| RMDQ | 0·165 (0·093, 0·243) |
| NPRS | 0·081 (0·031, 0·140) |
| DRS | 0·101 (0·047, 0·170) |
| EQ-5D | 0·000 (0·000, 0·056) |
| PSEQ-2 | 0·064 (0·000, 0·147) |
| Fear avoidance | 0.037 (0.000, 0.108) |
| Recovery expectation at 4w | 0·034 (0·000, 0·104) |
| Recovery expectation at 3m | 0·024 (0·000, 0·089) |
| Anxiety | 0·072 (0·003, 0·155) |
| Catastrophisation | 0·148 (0·053, 0·256) |
| RMDQ, Roland Morris Disability Questionnaire; NPRS, numeric pain rating scale; DRS, disability rating scale; EQ-5D, EuroQoL-5D; PSEQ-2, two item pain self-efficacy questionnaire | |

#### Discussion

Sample size planning for the study used a projected ICC of 0·05 for the primary outcome (Roland Morris Disability Questionnaire; RMDQ); the estimated ICC from within the study data was considerably higher at 0·165. This suggests that the final study result was subject to more sampling variability from the clustered design than initially anticipated; however, this uncertainty is correctly reflected in the reported confidence intervals in the study.

### Change in patient eligibility criteria primary outcome sensitivity analysis

Eligibility criteria changed early during the data collection phase (updated in the Trial registration and presented in the published protocol^2^). The protocol originally stated that the patient group would be limited to those who had experienced LBP for less than 6 weeks, were between 18 and 65 years, and had not seen a health professional about back pain in the last 3 months. It was thought that these criteria represented characteristics of people most likely to benefit from the FREE approach. However, these criteria were removed early in the study as these unnecessarily restricted patient eligibility and risked reducing the generalisability of findings.

Initial patient eligibility criteria

Patient participants were included if they:

- were between 18 and 65 years of age
- had LBP (with or without leg pain) of less than 6 weeks duration as their main reason for consultation

Patient participants were excluded if:

- had received consultation or treatment for this episode or for other LBP in the previous 3 months
- had back pain due to a non-back related condition (e.g. hip arthritis) or a serious health condition (e.g. cauda equina syndrome, spinal infection)
- had a concomitant health condition that meant they were unsuitable for trial participation (e.g. pregnancy or major psychological disturbance)
- were unable to read or write in English.

Final patient eligibility criteria (implemented from 1 January 2017)

Patient participants were included if they:

- were over 18 years of age
- had LBP of any duration (with or without leg pain) as their main reason for consultation

Patient participants were excluded if they:

- had received back surgery in the previous 6 months
- had been unable to do their normal work for more than 3 of the last 6 months
- had back pain due to a non-back related condition (e.g. hip arthritis) or a serious health condition (e.g. cauda equina syndrome, spinal infection)
- had a concomitant health condition that meant they were unsuitable for trial participation (e.g. pregnancy or major psychological disturbance)
- were unable to read or write in English.

#### Method

To explore the impact of the change in eligibility criteria, we conducted a sensitivity analysis for the primary outcome (RMDQ) restricted to those meeting the original eligibility criteria. This meant excluding individuals who were only eligible under the revised criteria. These were for: those aged over 65 (n=12 total; n=11 removed with follow-up data); those with back pain of more than 6 weeks duration (n=21; n=19 removed with follow-up data); or those having current or recent treatment for this back pain from another health professional (n=20 removed, n=20 with follow-up data). Some participants were excluded based on meeting multiple criteria: a total of 40 additional patients were excluded (compared to the analysis reported in main body of report) leaving a sample size of 171 patients meeting the original eligibility criteria and having at least one follow-up RMDQ measure. All other elements of the analysis followed the methods described in the main article.

#### Result

Estimates of effect size were almost identical to the main results (see Figure S1 and Table S4) and did not affect the main conclusions drawn (that the FREE intervention returns average improvements by 6 months that are not substantively different from practice as usual).


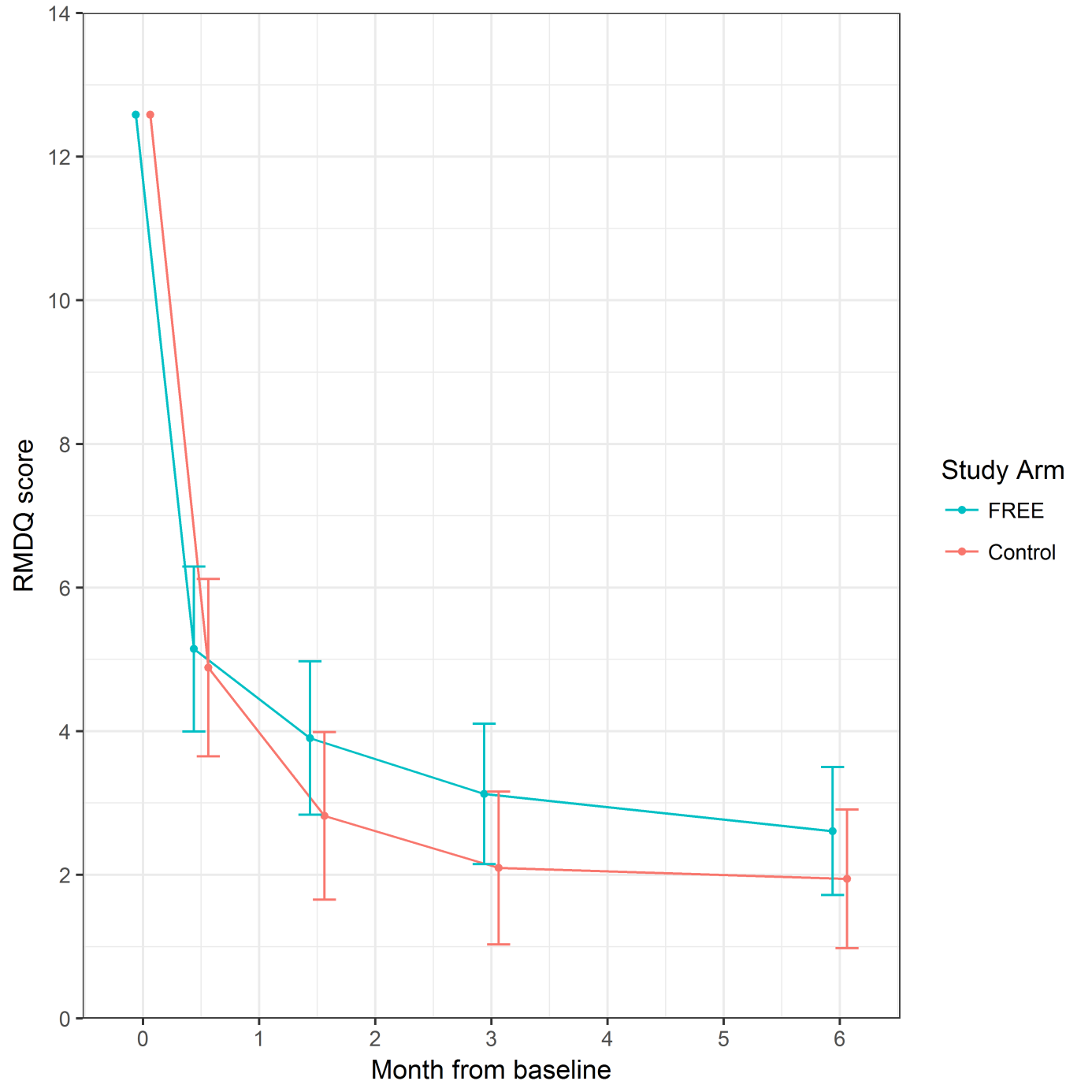


**Figure S1**. Sensitivity analysis restricted to originally eligible sample. Mean Roland Morris Disability Questionnaire score at baseline and follow-up (error bars give 95% CI of mean). Scale range 0 to 24, higher scores indicate higher levels of functional impairment

| ***Table S4.* Mean differences in primary outcome between groups at 2 weeks, 6 weeks, 3 months, and 6 months using final eligibility criteria and original eligibility criteria** | | | | | | |
| --- | --- | --- | --- | --- | --- | --- |
|  | **Mean difference (95% CI) at follow-up (95%CI)*** | | | | **Number of participants contributing data to analysis by outcome**  **(number of GPs with patients)** | |
| **Outcome** | **2 weeks** | **6 weeks** | **3 months** | **6 months** | **Intervention** | **Control** |
| **Primary outcome (final eligibility criteria†)** | | |  |  | 122 (32) | 99 (25) |
| RMDQ | –0·08 (–1.56, 1.41) | 0.79 (–0·62, 2·20) | 0·80 (–0·51, 2·12) | 0·57 (–0.64, 1·78) |  |  |
| **Primary outcome (original eligibility criteria‡)** | | |  |  | 91 (28) | 80 (25) |
| RMDQ | 0·26 (–1·45, 1·97) | 1·08 (–0·52, 2·69) | 1·03 (–0·44, 2·50) | 0·67 (–0.68, 2·.01) |  |  |
| *Positive scores indicate higher score in FREE arm than control arm; negative scores indicate higher score in control arm than FREE arm. Lower RMDQ values represent better outcomes. † Result is as per main results table. **‡**Excluding individuals aged over 65 (n=12 total; n=11 removed with follow-up data); with back pain of more than 6 weeks duration (n=21; n=19 removed with follow-up data); or having current or recent treatment for this back pain from another health professional (n=20 removed, n-20 with follow-up data) | | | | | | |

### Sensitivity analysis for primary outcome: participants missing baseline co-variates

#### Method

A total of 15 participants were missing baseline data (on one or more of the pain self-efficacy items, expectation of recovery at 4 weeks, and expectation of recovery at 3 months)(Table S5). These data were missing as participants had been unable to complete all paperwork before seeing their GP, and so it was assumed that the data were missing completely at random and unlikely to bias the results. Mean imputation was used to be able to include all participants with follow-up data in the main analysis. ^3^

Since the protocol had not specified how missing baseline covariate data would be handled, we also conducted two sensitivity analyses for the primary outcome. The first sensitivity analysis excluded those people with missing data for one or more baseline covariates (n=15); the second sensitivity analysis included all these participants (as per the main model) but did not adjust for the three baseline covariates with missing data.

| ***Table S5*. Data missing at baseline** | | |
| --- | --- | --- |
| **Patient Characteristic** | **FREE (n=126)** | **Control (n=100)** |
| RMDQ | 0 | 0 |
| NPRS Back | 3 | 8 |
| NPRS Leg | 4 | 9 |
| DRS | 3 | 8 |
| EQ-5D | 5 | 10 |
| PSEQ-2 | 13 | 13 |
| Recovery expectations |  |  |
| 4 weeks | 5 | 10 |
| 3 months | 5 | 10 |
| Fear avoidance | 5 | 9 |
| Anxiety | 5 | 9 |
| Catastrophisation | 6 | 11 |
| RMDQ, Roland Morris Disability Questionnaire; NPRS, numeric pain rating scale; DRS, disability rating scale; EQ-5D, EuroQoL-5D; PSEQ-2, two item pain self-efficacy questionnaire | | |

#### Results

Table S6 presents results from the three sensitivity analyses. While point estimates differed slightly between models, these differences were insubstantial and had no impact on the conclusions drawn in the main analysis presented in the paper.

| ***Table S6.* Mean differences in primary outcome between groups at 2 weeks, 6 weeks, 3 months, and 6 months using final eligibility criteria and original eligibility criteria** | | | | | | |
| --- | --- | --- | --- | --- | --- | --- |
|  | **Mean difference (95% CI) at follow-up (95%CI)*** | | | | **Number of participants contributing data to analysis**  **(number of GPs with patients)** | |
| **Outcome** | **2 weeks** | **6 weeks** | **3 months** | **6 months** | **Intervention** | **Control** |
| **Primary outcome (as per main results†)** | | |  |  | 122 (32) | 99 (25) |
| RMDQ | –0⋅08 (–1.56, 1.41) | 0.79 (–0⋅62, 2⋅20) | 0⋅80 (–0⋅51, 2⋅12) | 0⋅57 (–0·64, 1⋅78) |  |  |
| **Excluding patients with missing baseline covariate data‡** | | | |  | 117 (31) | 89 (25) |
| RMDQ | –0⋅05 (–1⋅63, 1⋅52) | 0⋅82 (–0⋅68, 2⋅31) | 0⋅81 (–0⋅59, 2⋅22) | 0⋅66 (–0·65, 1⋅96) |  |  |
| **Omitting covariates with any missing baseline (less adjusted)** **§** | | | | | 122 (32) | 99 (25) |
| RMDQ | –0⋅07 (–1⋅68, 1⋅54) | 0⋅78 (–0⋅74, 2⋅31) | 0⋅79 (–0⋅62, 2⋅20) | 0⋅52 (–0·74, 1⋅77) |  |  |
| *Positive scores indicate higher score in FREE arm than control arm; negative scores indicate higher score in control arm than FREE arm. Lower RMDQ values represent better outcomes. † Result is as per main results table. **‡** Model excluding participants with missing data on one or more baseline covariates. § Model omitting adjustment for baseline covariates with any missing data (PSEQ2, Expectation of recovery at 4 weeks and Expectation of recovery at 3 months) | | | | | | |

### Number Needed to Treat Primary Outcome Analysis

In the protocol, we specified that for the primary outcome we would report the relative risk of a 30% improvement in RMDQ scores along, with the numbers needed to treat (NNT) to achieve this target.^4^ As a sensitivity analysis, the NNT and RR of a 2·5 point reduction in RMDQ score were also specified (matching the absolute effect size stipulated for the primary outcome).

The NNT would then represent the number of patients needed to be treated with FREE (rather than practice as usual) to return a clinically important benefit for one patient. As per the above note, “benefit” was pre-specified as a 30% improvement in outcome from baseline to six months (e.g. for a patient with the sample-mean score of around 13 points at baseline, this would be an improvement of 3·8 points by six months).

#### Method

As the first step in this process we calculated the absolute difference in proportions of patients achieving an improvement of at least this size. The majority of patients achieved at least a 30% improvement in RMDQ score (FREE arm: n = 101/116, or 87·1%; Control arm, n = 86/96 patients, or 89·6%). This equates to an absolute difference of –2·5 percentage points (95% CI –11·2, 6·1) and an RR of 0·97 (95% CI 0·88, 1·07; relative difference in proportion achieving 30% improvement) with lower achievement in the FREE arm.

As FREE did not return a statistically significant improvement over the control arm on RMDQ score (either on the continuous measure or as the proportion of patients achieving a sizeable improvement), the NNT is not straightforward to interpret: this is because the confidence interval for an NNT in this situation technically spans infinity (that is, if there is truly “no difference at all” between FREE and control then it is impossible to achieve one patient gain regardless of how many patients are treated). It has been recommended that studies with non-significant results do not report an NNT to avoid this complexity.^5^ The details of the calculations are given below.

The NNT was calculated based on the odds ratio derived from a logistic regression model for the relative difference in achieving a 30% improvement by study arm, adjusted for practice size strata as the sole adjustment variable. This was conducted following the methods outlined in Bender et al.^6^ (estimates were broadly similar when calculated from an unadjusted absolute risk reduction).

#### Results

As noted earlier, since this comparison returns a confidence interval that spans the null result (no difference in treatment outcomes) the confidence interval for the NNT ranges from a net benefit of FREE (upper bound) down to a net detriment of FREE (lower bound) relative to Control treatment (referred to as number needed to harm, or NNH, with the FREE approach).

This returned an NNH of 45·2 patients: that is, for every 45 patients treated with FREE, we would see one less patient improvement (of 30% or more of initial RMDQ score) than seen under control treatment.

The upper bound of the 95% confidence interval (i.e. the most optimistic estimate for FREE) was a NNT of 21·7 (that is, for every 22 patients treated with FREE, you would see one additional improvement over the control treatment.) The lower bound of the 95% confidence interval (i.e. the least optimistic estimate for FREE) was a number needed to harm (NNH) of 6·7 – that is, for every 7 patients treated with FREE, we would see one less patient with an improvement than treatment under control.

To summarise: from an NNT point of view the true impact likely falls between control doing better than FREE (NNH of 6·7) through equivalence (no impact of treatment type on outcomes) to FREE doing better than control (NNT of 21·7). This is consonant with the main findings of the study the report.

The protocol had also stated that we would calculate and report the NNT for people achieving a reduction of at least 2·5 points on the RMDQ: however, all but two people met this criteria (across both arms), and so the RR and NNT could not be calculated.

### Patient satisfaction

#### Method

Patient satisfaction with information and care received and treatment results was measured with 7 point Likert scales (Table S7).^7 8^ Week 0 satisfaction items were included in the brief survey that patients were asked to complete immediately following their initial GP consultation for LBP (before leaving the medical practice).

These categorical outcomes were compared using generalised linear mixed models. These models adjusted for important baseline covariates (age, gender, socioeconomic status, current back pain duration and nature (constant or episodic), receipt of recent or ongoing non-GP health care for back pain, previous history of back pain, baseline disability, and baseline psychological factors (pain-self-efficacy and recovery expectations)).^9^

| ***Table S7.* Patient satisfaction measures and time points at which these were assessed.** | |
| --- | --- |
| **Satisfaction item** | **Data collection time points** |
| With information received  How satisfied are you with the information you received about your back pain?  Where 0 = very dissatisfied and 6 = very satisfied | 0, 2w, |
| With care received ^7^ modified by ^8^  How satisfied are you with the treatment you received for your back pain?  Where 0 = very dissatisfied and 6 = very satisfied | 0, 2w, 6w, 6m |
| Rating of the overall results of care ^7^  If you had to spend the rest of your life with the symptoms you have right now, how would you feel about it?  Where 0 = very dissatisfied and 6 = very satisfied | 2w, 6w, 6m |

#### Results

Patient satisfaction immediately post-appointment with the information and care received at the recruitment appointment was slightly different between study arms (Table S8 gives the full response category breakdown). Those in the FREE arm were slightly less likely to be satisfied with the information received (combined somewhat and very satisfied responses: 86·9% for FREE arm, 95·0% for Control arm) and with care received more broadly (86·5% for FREE, 94·0% for Control).

Table S9. presents trajectories of satisfaction ratings over the study period as measured immediately post-appointment, at 2 weeks and at 6 weeks (at 6 weeks: only satisfaction with care was assessed) and also includes inferential statistics formally testing whether satisfaction (measured as an ordinal outcome) was higher in the FREE or Control arm. These tests indicated that at week 0 (immediately after the appointment) patients in the FREE arm were more likely to give a lower “satisfaction with information received” rating than in the Control arm (p = 0·047). While rating profiles in the FREE arm were lower than in Control for satisfaction with information ratings at week 2 and satisfaction with care ratings at weeks 0, 2, and 6, the statistical evidence for these differences, and for differences in satisfaction with overall results of care (measured at 2, 6 and 26 weeks), was limited (p-values ranging from 0.100 to 0.867; see Table S9 for details).

| ***Table S8*. Patient reported satisfaction immediately post-appointment.** | | | | |
| --- | --- | --- | --- | --- |
| **Satisfaction variable** | **Response** |  | **% reporting recommendation (95% CI)** | |
|  |  |  | **FREE (n=126,  32 GPs)** | **Control (n=100,  25 GPs))** |
| Satisfaction with | Very dissatisfied |  | 4·0 (1·7, 8·9) | 1·0 (0·1, 7·5) |
| information received | Somewhat dissatisfied |  | 3·2 (1·2, 8·1) | 2·0 (0·5, 8·0) |
|  | Neither satisfied nor dissatisfied |  | 6·3 (3·1, 12·5) | 2·0 (0·4, 8·5) |
|  | Somewhat satisfied |  | 24·6 (17·8, 33·0) | 21·0 (12·1, 34·0) |
|  | Very satisfied |  | 61·9 (51·6, 71·2) | 74·0 (61·9, 83·3) |
| Satisfaction with | Very dissatisfied |  | 4·8 (2·3, 9·7) | 1·0 (0·1, 7·5) |
| care received | Somewhat dissatisfied |  | 2·4 (0·8, 7·2) | 1·0 (0·1, 7·3) |
|  | Neither satisfied nor dissatisfied |  | 6·3 (3·3, 11·8) | 4·0 (1·5, 10·5) |
|  | Somewhat satisfied |  | 26·2 (19·2, 34·6) | 25·0 (16·9, 35·3) |
|  | Very satisfied |  | 60·3 (50·6, 69·3) | 69·0 (57·7, 78·4) |

| ***Table S9.* Satisfaction ratings for information and care received over the follow-up period, by study arm.** | | | | | | | | | | | | | |
| --- | --- | --- | --- | --- | --- | --- | --- | --- | --- | --- | --- | --- | --- |
|  |  | **Week 0 (after appointment)** | | | **Week 2** | | | **Week 6** | | | **Week 26** | | |
|  |  | **FREE** | **Control** | **Ordinal** | **FREE** | **Control** | **Ordinal** | **FREE** | **Control** | **Ordinal** | **FREE** | **Control** | **Ordinal** |
| **Satisfaction** | **Level** | **n (%)** | **n (%)** | **p-value*** | **n (%)** | **n (%)** | **p-value*** | **n (%)** | **n (%)** | **p-value*** | **n (%)** | **n (%)** | **p-value*** |
| Satisfaction |  | n=126 (32) | n=100 (25) |  | n=111 (31) | n=88 (24) |  |  |  |  |  |  |  |
| with | 1 | 5 (4.0) | 1 (1.0) |  | 1 (0.9) | 3 (3.4) |  |  |  |  |  |  |  |
| information | 2 | 4 (3.2) | 2 (2.0) | 0.047 | 13 (11.7) | 3 (3.4) | 0.867 |  |  |  |  |  |  |
|  | 3 | 8 (6.3) | 2 (2.0) |  | 16 (14.4) | 11 (12.5) |  |  |  |  |  |  |  |
|  | 4 | 31 (24.6) | 21 (21.0) |  | 33 (29.7) | 38 (43.2) |  |  |  |  |  |  |  |
|  | 5 | 75 (59.5) | 74 (74.0) |  | 48 (43.2) | 33 (37.5) |  |  |  |  |  |  |  |
| Satisfaction |  | n=126 (32) | n=100 (25) |  | n=111 (31) | n=87 (24) |  | n=113 (31) | n=87 (24) |  | n=115 (31) | n=93 (24) |  |
| with care | 1 | 6 (4.8) | 1 (1.0) |  | 4 (3.6) | 4 (4.5) |  | 6 (4.8) | 1 (1.0) |  | 7 (6.1) | 6 (6.5) |  |
|  | 2 | 3 (2.4) | 1 (1.0) | 0.100 | 8 (7.2) | 5 (5.7) | 0.301 | 11 (8.7) | 8 (8.0) | 0.230 | 11 (9.6) | 7 (7.5) | 0.662 |
|  | 3 | 8 (6.3) | 4 (4.0) |  | 21 (18.9) | 6 (6.8) |  | 24 (19.0) | 9 (9.0) |  | 25 (21.7) | 11 (10.8) |  |
|  | 4 | 33 (26.2) | 25 (25.0) |  | 34 (30.6) | 36 (40.9) |  | 30 (23.8) | 32 (32.0) |  | 21 (18.3) | 32 (35.5) |  |
|  | 5 | 76 (60.3) | 69 (69.0) |  | 44 (39.6) | 36 (40.9) |  | 42 (33.3) | 37 (37.0) |  | 51 (44.3) | 37 (39.8) |  |
| Rating of the |  |  |  |  | n=110 (31) | n=88 (24) |  | n=113 (31) | n=87 (24) |  | n=115 (31) | n=93 (24) |  |
| overall | 1 |  |  |  | 54 (49.1) | 44 (50.0) |  | 45 (39.8) | 25 (28.7) |  | 28 (24.3) | 23 (24.7) |  |
| results | 2 |  |  |  | 18 (22.7) | 18 (20.5) | 0.902 | 24 (21.2) | 19 (21.8) | 0.317 | 29 (25.2) | 23 (24.7) | 0.747 |
| of care | 3 |  |  |  | 11 (6.4) | 11 (12.5) |  | 11 (9.7) | 16 (18.4) |  | 14 (12.2) | 9 (9.7) |  |
|  | 4 |  |  |  | 11 (11.8) | 11 (12.5) |  | 13 (11.5) | 17 (19.5) |  | 17 (14.8) | 15 (16.1) |  |
|  | 5 |  |  |  | 4 (10.0) | 4 (4.5) |  | 20 (17.7) | 10 (11.5) |  | 27 (23.5) | 23 (24.7) |  |
| * p-value for hypothesis test for having a higher level of satisfaction in FREE arm relative to Control arm (as an ordinal outcome). Calculated in mixed-effects ordinal logistic regression, accounting for repeated measures outcome and clustering by GP. Estimates are adjusted for age, prioritised ethnicity, NZDep decile, length of back pain, consistency of back pain, previous back pain, patient self-efficacy, recovery expectations (4 weeks and 4 months), GP’s HC-PAIRS score, and practice size stratum. | | | | | | | | | | | | | |

### Psychosocial process measures

People are more likely to present to health care services with LBP when they have high levels of disability, pain or psychosocial distress.^10 11^ These co-existent factors are associated with increased risk of developing persistent disabling LBP.^12 13^

#### Method

To examine mechanisms by which FREE might impact on patient outcomes we collected patient reported psychosocial measures (fear avoidance,^14 15^ pain self-efficacy,^16^ catastrophisation,^17^ anxiety,^14^ and expectation^18 19^) at baseline, 2 weeks and 6 weeks (the normal recovery period for most LBP). These items are presented in Table S10. Some items were modified for consistency (use of first person in questions for self-report, and Likerts scales all set to a 7-point range).

| ***Table S10.* Psychosocial process measures and time points at which these were collected** | | |
| --- | --- | --- |
| **Domain** | **Instrument** | **Data collection time points** |
| Pain self-efficacy | PSEQ-2 ^16^  Please rate how **confident** you are that you can do the following things at present, **despite the pain**   1. I can do some form of work, despite the pain (‘‘work’’ includes housework and paid and unpaid work). 2. I can live a normal lifestyle, despite the pain   where 0 = not at all confident and 6 = completely confident | 0, 2w, 6w, |
| Expectation | 1. I think I will be able to do my regular job, without any restrictions, 4 weeks from now ^18^ 2. I think I will be able to do my regular job, without any restrictions, 3 months from now ^19^   Where 0 = Unlikely and 6 = Definitely | 0, 2w, 6w |
| Fear avoidance | 1. Physical activity might harm my back 2. I should not do physical activities which (might) make my pain worse ^14 15^   0 = Completely disagree and 6 = Completely agree | 0, 2w, 6w |
| Anxiety | 1. I feel anxious or worried about my back^14^   0 = Not at all and 6 = Quite anxious | 0, 2w, 6w |
| Catastrophisation | 1. I feel that my back pain is terrible and it’s never going to get any better ^17^   Where 0 = Not at all and 6 = All the time | 0, 2w, 6w |

These outcomes were analysed using linear mixed models adjusted for important baseline covariates (age, gender, socioeconomic status, current back pain duration and nature (constant or episodic), receipt of recent or ongoing non-GP health care for back pain, previous history of back pain, baseline disability, and baseline psychological factors (pain-self-efficacy and recovery expectations)).^9^

#### Results

For each of the mediator outcomes, mean scores tended to be better for the FREE arm than the control arm at 2 weeks (Table S11; Figure S2). While this was consistent across outcomes, for most mediator variables the confidence intervals indicated the potential for no difference between study arms (Table S11: e.g. anxiety at 2 weeks: mean difference = –0·45, 95% CI –0·98 to 0·08). The exception to this was fear avoidance, which was significantly lower in the FREE than control arm at 2 weeks (mean difference = –0·65,–1·16 to –0·14; p = 0·013).

The magnitudes of mean differences in these outcomes were smaller at 6 weeks than at 2 weeks, and confidence intervals indicated high potential for no real differences by study arm (e.g. fear avoidance mean difference at 6 weeks = –0·22, –0·75 to 0·32).

| ***Table S11.* Mean differences (95% CI) in patient moderator outcomes at each follow-up time, and number of patients contributing data to analysis for each outcome.** | | | | |
| --- | --- | --- | --- | --- |
| **Outcome** | **Mean difference (95% CI) at follow-up (FREE - Control)*** | | **n patients contributing data to analysis by outcome** | |
|  | **t=2 weeks** | **t=6 weeks** | **(FREE)**  **n=126, 30 GPs** | **(Control)  n =100, 24 GPs** |
| Pain Self Efficacy (PSEQ-2) † | 0·37 (-0·11, 0·85) | –0·21 (–0·64, 0·22) | 112 | 82 |
| Fear avoidance**‡** | –0·65 (-1·16, -0·14) | –0·22 (–0·75, 0·32) | 112 | 83 |
| Recovery expectation at 4w† | 0·37 (–0·11, 0·85) | 0·30 (–0·20, 0·81) | 112 | 82 |
| Recovery expectation at 3m† | 0·22 (–0·20, 0·64) | 0·35 (–0·15, 0·84) | 112 | 82 |
| Anxiety**‡** | –0·45 (–0·98, 0·08) | –0·25 (–0·81, 0·31) | 112 | 83 |
| Catastrophisation**‡** | –0·29 (–0·74, 0·15) | –0·03 (–0·59, 0·53) | 111 | 81 |
| PSEQ-2, two item pain self-efficacy questionnaire. *Positive scores indicate higher score in FREE arm than control arm; negative scores indicate higher score in control arm than FREE arm. † higher scores indicate enhanced positive influences of psychological facilitators of recovery. **‡** lower scores indicate reduced negative influences of psychological barriers to recovery. | | | | |

**
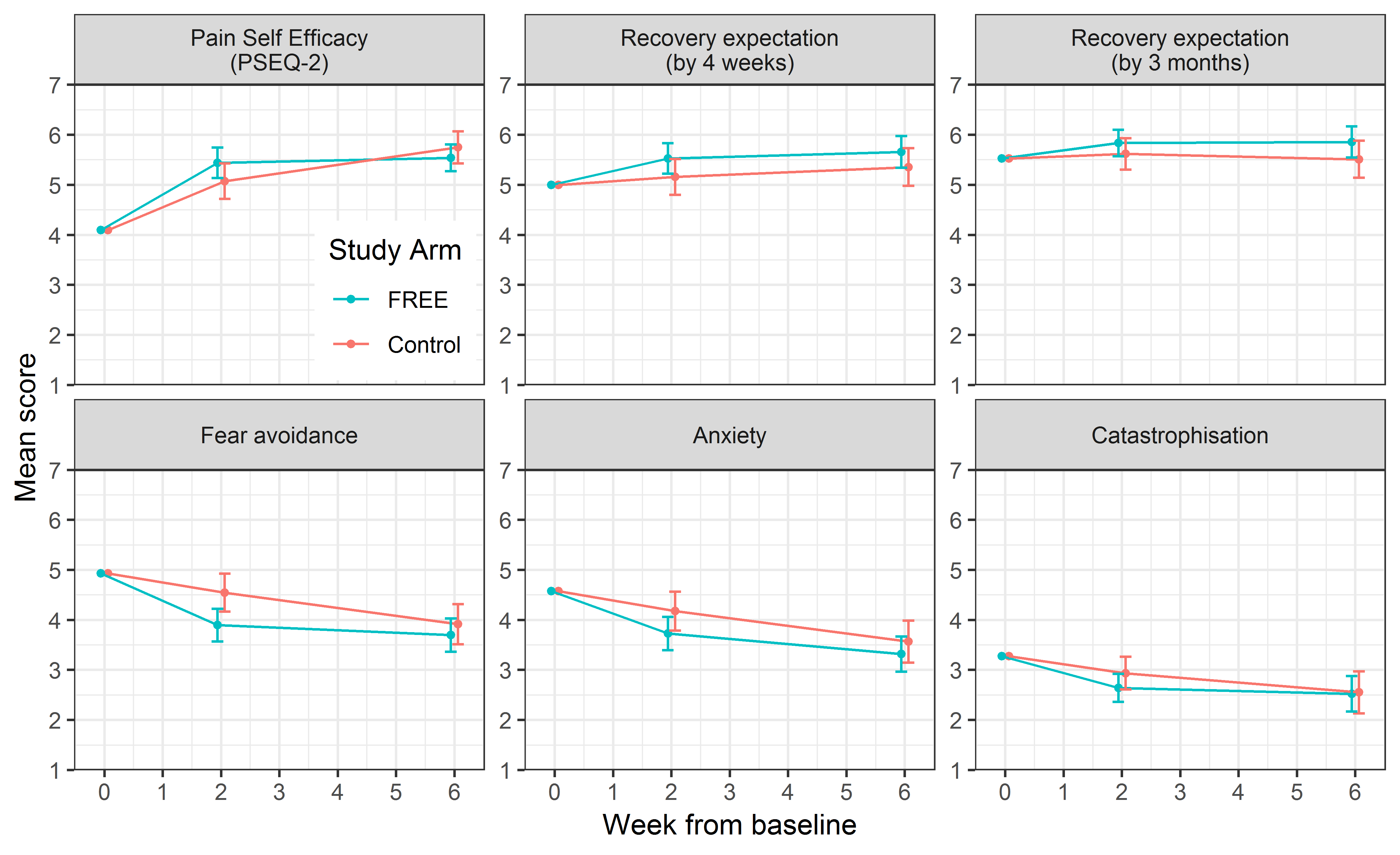
**

**Figure S2**. **Mean outcomes for patient-level mediator variables at baseline, 2 and 6 weeks.** **Top-row**: higher scores indicate enhanced positive influences of psychological facilitators of recovery; PSEQ-2, two item pain self-efficacy questionnaire. **Bottom row**: lower scores indicate reduced negative influences of psychological barriers to recovery. Error bars indicate 95% CI.

## Harms

#### Methods

This study employed both active and passive harm surveillance. Patients and GPs were encouraged to report all potential adverse events and incidents of serious pathology (and method of discovery) amongst trial participants to the research team on a continuous basis during the study. In addition, patients were encouraged to record adverse events in their study log book. Active surveillance of harms occurred through the final page of each study survey (GP and patient) requesting reports of unexpected or serious health events using a standardised form. The patient participant form is presented below (Figure S3).

Each report was investigated by an investigator (TD) who was an experienced GP and blinded to group allocation using a standardised Adverse Event Reporting Form. The investigator judged whether the event was: i) expected or unexpected; ii) potentially related to the back; iii) potentially related to the intervention; and iv) mild, moderate or serious. Serious adverse events were defined (as per National Ethics Advisory Committee guidelines) as untoward medical occurrences as a result of the intervention that^20^:

- Result in death, or
- Are life threatening, or
- Require inpatient hospitalisation or results in prolongation of existing hospitalisation, or
- Result in persistent or significant disability or incapacity, or
- Are medically important events or reactions

Each Adverse Event Reporting Form was reviewed by an independent academic GP who was a member of the data monitoring committee and approved by the entire data monitoring committee.

Harms were analysed descriptively and are presented in accordance with the CONSORT extension for reporting harms.^21^

#### Results

GP participants did not report any adverse events or incidents of serious pathology during the study. Patient participants reported 55 health events that they considered to be serious or unexpected and were investigated as potential adverse events (Table S12). Of these 55 reports, 26 were classified as minor (for example, flu-like illness or tripping), 24 were classified as moderate (for example, adverse reactions to medication unrelated to the intervention), and 5 were classified as serious (for example, pulmonary emboli). These reports were the mechanism through which three participants were excluded from the study as it became clear that they met exclusion criteria related to their back pain being due to a non-back-related condition (spinal infection, right and left common iliac artery stenosis, pulmonary embolism). Only one report was potentially related to the study intervention. This was a gluteus minimus tear sustained while dancing that was classified as moderate. A sports physician thought this was due to general lumbopelvic instability. This participant was in the control group; consequently, this event cannot have been related to the intervention. There were no serious adverse events.


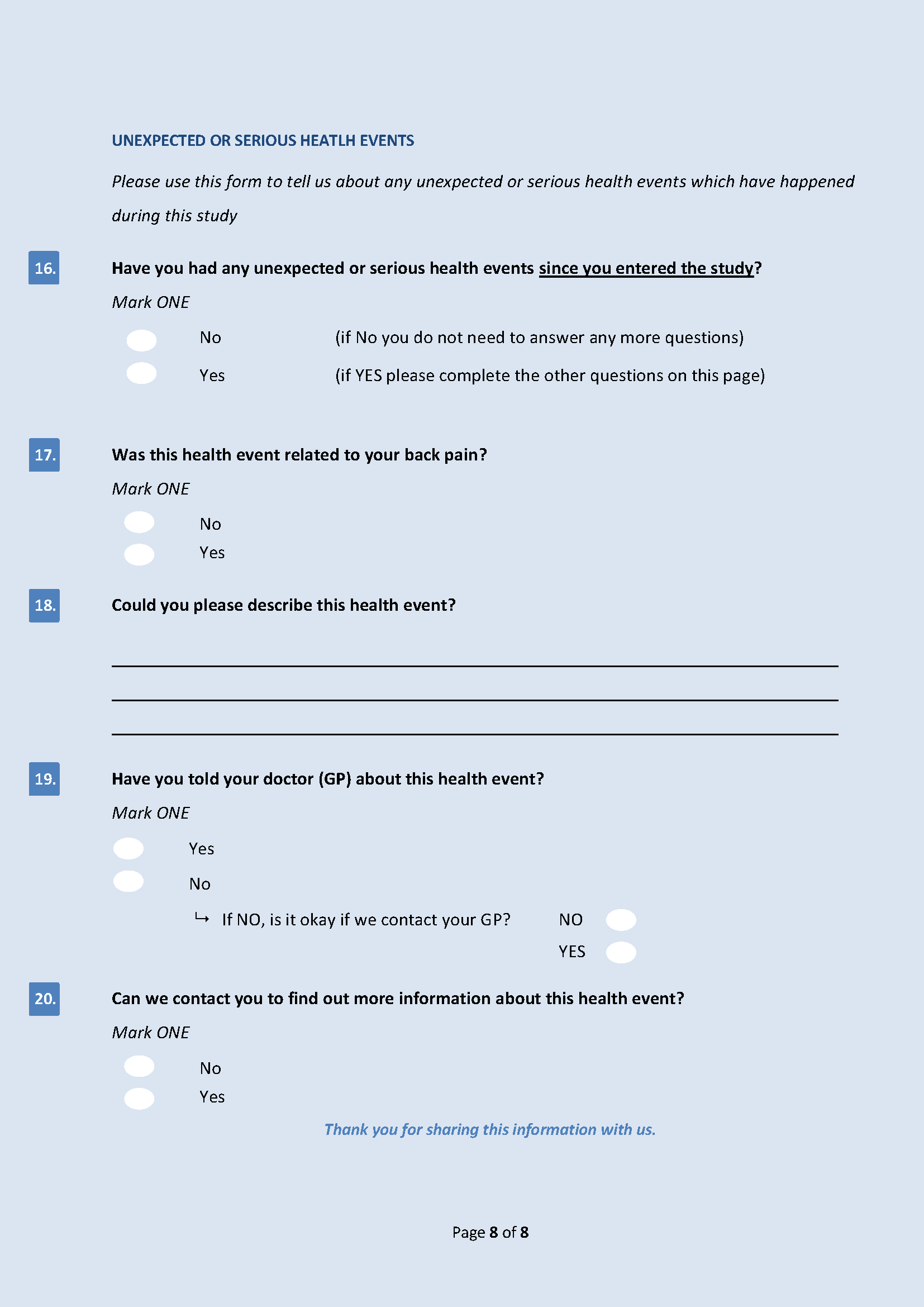


***Figure S3*. Standardised patient participant health event reporting form included in each study survey.**

| ***Table S12*. Summary of health events reported during the randomised clinical trial that were investigated as potential adverse events.** | | | | | | | |
| --- | --- | --- | --- | --- | --- | --- | --- |
| **Classific-ation** | **n** | **Group** | **Potentially back related** | **Potentially study related** | **Serious adverse event** | **Description** | **Outcome** |
| **Mild**  N = 26 | 9 | CONTROL | Yes - 1 | 0 | 0 | - Exacerbation of back pain when brushing teeth caused participant to twist her neck |  |
|  |  |  | No - 8 | 0 | 0 | - Flu-like illness (3) - Respiratory symptoms (1) - Joint sprain (1) - High blood pressure (1) - Broken tooth (1) - Recurrence of mental health symptoms (1) |  |
|  | 17 | FREE | Yes - 3 | 0 | 0 | - Longstanding shoulder pain increase after onset of back pain - Unsteady because not wearing back support. Tripped and fell on to knees - Lost balance due to back. Fell and hurt both knees. |  |
|  |  |  | No – 14 | 0 | 0 | - Other musculoskeletal pain (5) - Rectal bleed (1) - Flu like illness (2) - Respiratory symptoms (3) - Low vitamin B12 (1) - Amenorrhea (1) - Gastrointestinal infection (1) |  |
| **Moderate**  N = 24 | 10 | CONTROL | Yes -3 | Yes -1 | 0 | - Gluteus minimus tear sustained while dancing. Sports physician thought was due to general lumbopelvic instability |  |
|  |  |  |  | No - 2 | 0 | - Fainted after taking codeine, Naproxen and tramadol and hit head - Tramadol caused flare of IBS symptoms (constipation and rectal bleeding) |  |
|  |  |  | No – 7 | 0 | 0 | - Fractured rib as a result of fall - Twisted Left knee, query meniscus damage - Severe migraines x 2 requiring ED attendance - Injured thumb, required surgery - Had a fall, passed out, then experienced chest pain two days later - Slipped down stairs – multiple bruising - Subluxed foot during fall |  |
|  | 14 | FREE | Yes – 1 | No - 1 |  | - Onset of numbness in legs |  |
|  |  |  | No – 13 |  |  | - Allergic reaction - Gastro-intestinal (3) - Musculoskeletal injury (3) - Mental health issue - Fall - Bacterial/Viral infection (2) - Respiratory diagnosis - Pericarditis |  |
| **Serious**  N= 5 | 1 | CONTROL | Yes -1 | 0 | 0 | - Spinal Infection | Withdrawn |
|  | 4 | FREE | Yes - 1 | 0 | 0 | - Vertebral fractures x 2 |  |
|  |  |  | No - 3 |  |  | - Right and left common iliac stenosis - Pulmonary embolism - Requires hip replacement | Withdrawn Withdrawn |

##

## GP outcomes

### GP satisfaction with training

#### Method

Satisfaction with training amongst intervention arm GPs was measured with a bespoke questionnaire that included Likert scales and free-text response options. These were analysed with descriptive statistics.

#### Result

Workshop evaluations were completed by 31 GPs in the FREE arm. These GPs evaluated the initial training workshop highly (Figure S4).


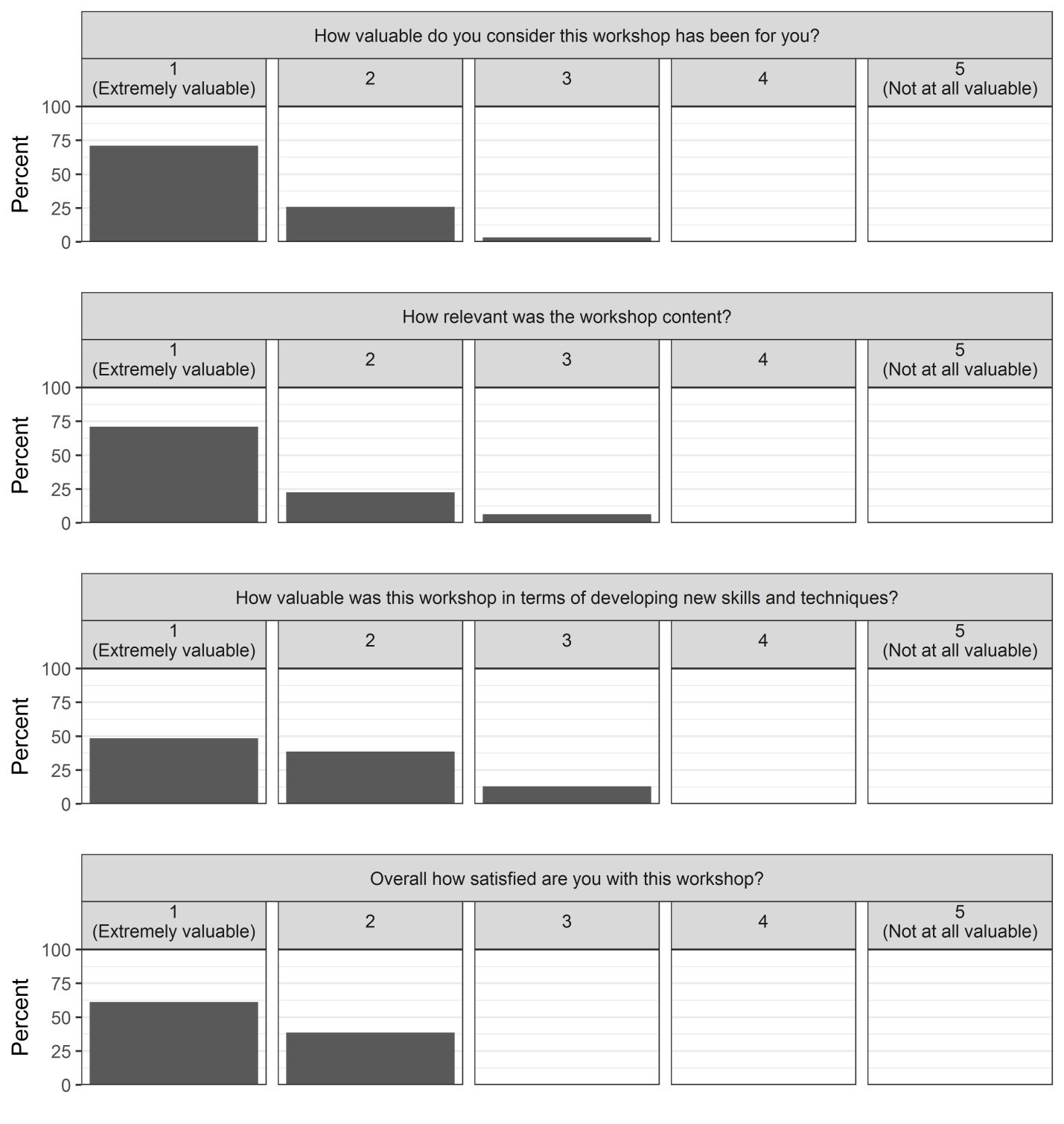


**Figure S4. Response distribution to Items from FREE training workshop evaluation forms**

### Patient report of GP management recommendations

#### Method

Immediately following their initial GP consultation for LBP (before leaving the medical practice), patient participants were asked to complete a brief survey that included items related to GP management recommendations (Table S13).

Separate analyses were conducted for recommendations regarding work, activity, and medication, and for referrals to physiotherapy/osteopathy/chiropractic/acupuncture, specialist, X-rays or other scans. These analyses estimated the proportion of consultations showing these different outcomes, accounting for clustering by GP in the estimation of the confidence intervals.

| ***Table S13.* Patient report of GP management.** | |
| --- | --- |
| **Reported GP management item** | **Data collection time points** |
| Patient report immediately post appointment  Did your doctor recommend or advise any of the following? (yes or no)   - Time off work - Reduced hours at work - Medication - Physiotherapy/Osteopathy/Chiropractic - Specialist - X-rays or scans - Blood tests | 0w – obtained immediately after each participants’ LBP consultation |

#### Results

Patients’ self-reports of what their GP had recommended about work participation were similar between study arms (Table S14): roughly equal numbers of individuals reported being recommended to continue with work (as normal, or on reduced hours or restricted duties). Around one-quarter of patients reported being recommended to take time off work (26·3% for the FREE arm; 28·4% for the Control arm).

Activity recommendations were substantially different between study arms: a higher proportion of FREE patients reported being recommended to continue with normal activity (68·6%, 95% CI 60·8 to 75·5) than in the Control arm (43·7%, 32·9 to 55·0). Recommendations to avoid activity were relatively rare in both groups, but again lower in the FREE arm than the Control arm (1·7% and 8.0% respectively; Table 4.4.)

Referrals to physiotherapy/osteopathy/chiropractic/ acupuncture were substantially lower in the FREE arm (24·6%, 16·4 to 35·1) than in the Control arm (68·0%, 58·2 to 76·4). Referrals to specialists and for imaging were infrequent in both groups; smaller proportions of patients in the FREE arm than in the control arm reported referrals for imaging (7·9%, 4.4 to 13·8 and 11·0% 5·7 to 20·2 respective) and referral to specialists (2·4%, 0·8 to 7·2 and 6·0%, 2·9 to 12·1 respectively), however, confidence intervals for both of these referral categories overlapped.

| ***Table S14*. Patient reported GP management recommendations collected immediately post-appointment**. | | | | |
| --- | --- | --- | --- | --- |
| **Recommendation** | **Reported by patients** |  | **% reporting recommendation (95% CI)** | |
|  |  |  | **FREE (n=126,  32 GPs)** | **Control (n=100,  25 GPs)** |
| Work | Continuing with your normal work |  | 55·3 (43·7, 66·3) | 47·7 (37·9, 57·8) |
|  | Staying at work on reduced hours or restricted duties |  | 18·4 (12·1, 27·0) | 23·9 (15·7, 34·5) |
|  | Taking time off work |  | 26·3 (16·9, 38·5) | 28·4 (21·4, 36·6) |
| Activity | Continuing with your normal activity |  | 68·6 (60·8, 75·5) | 43·7 (32·9, 55·0) |
|  | Limiting or reducing your normal activity |  | 29·7 (23·7, 36·4) | 48·3 (36·6, 60·1) |
|  | Avoiding activity |  | 1·7 (0·4, 6·5) | 8·0 (4·3, 14·6) |
| Other treatment | Medication |  | 89·7 (80·4, 94·8) | 89·0 (82·3, 93·4) |
|  | Physiotherapist/osteopath/chiropractor/ |  | 24·6 (16·4, 35·1) | 68·0 (58·2, 76·4) |
|  | acupuncturist |  |  |  |
|  | Imaging |  | 7·9 (4·4, 13·8) | 11·0 (5·7, 20·2) |
|  | Specialist |  | 2·4 (0·8, 7·2) | 6·0 (2·9, 12·1) |

### Electronic consultation record audit

#### Methods

Consultation content entered by GPs in the electronic medical record was recorded by research nurses following each consultation using a structured template (Figure S5). Research nurses received training to identify these features and likely variations of reporting and abbreviations.

Separate analyses were conducted for each item. These analyses estimated the proportion of consultations showing these different outcomes, accounting for clustering by GP in the estimation of the confidence intervals (using complex survey estimation techniques, treating GP as the primary sampling unit: implemented in R package version 3.32).^22 23^

#### Results

Patients in the FREE study arm had a greater number of items consistent with best practice guidelines entered in their consultation record by their GP (explanation of back pain, providing reassurance and advice about moving) (Table S15).

Patients in the FREE arm were less likely to have consideration of red flags entered in their patient notes (42·4%, 95%CI 26.9 to 59.5, compared to 60·6%, 44·2 to 74·9 in the Control arm), but more likely to have patient concerns or worries (or the absence thereof) noted (33·6%, 23·3 to 45·8 compared to 1·0%, 0·1 to 7·7 in the Control arm).

Insurance claims to New Zealand’s national, no-fault accident and injury insurer, the Accident Compensation and Rehabilitation Corporation (ACC) were less commonly linked to the consultation for patients in the FREE arm (54·4%, 43·4 to 64·9) than in the Control arm (69·1%, 59·3 to 77·4).

Consistent with the patient report information, a lower proportion of patients in the FREE arm had referral to an adjunct health professional or specialist noted in the medical record (21·6%, 14·5 to 30·9) than the Control arm (49·5%, 39·1 to 55·9). Provision of a prescription for medication was similar in both arms (82·4 and 88·9%, respectively for FREE and Control arms). These data do not include informal referrals such as statements like “you could go and see a physiotherapist *if you like*,” or medication recommendations like “take some Panadol *if you like*.” This may explain the slight variation between these data and those reported directly from patients.


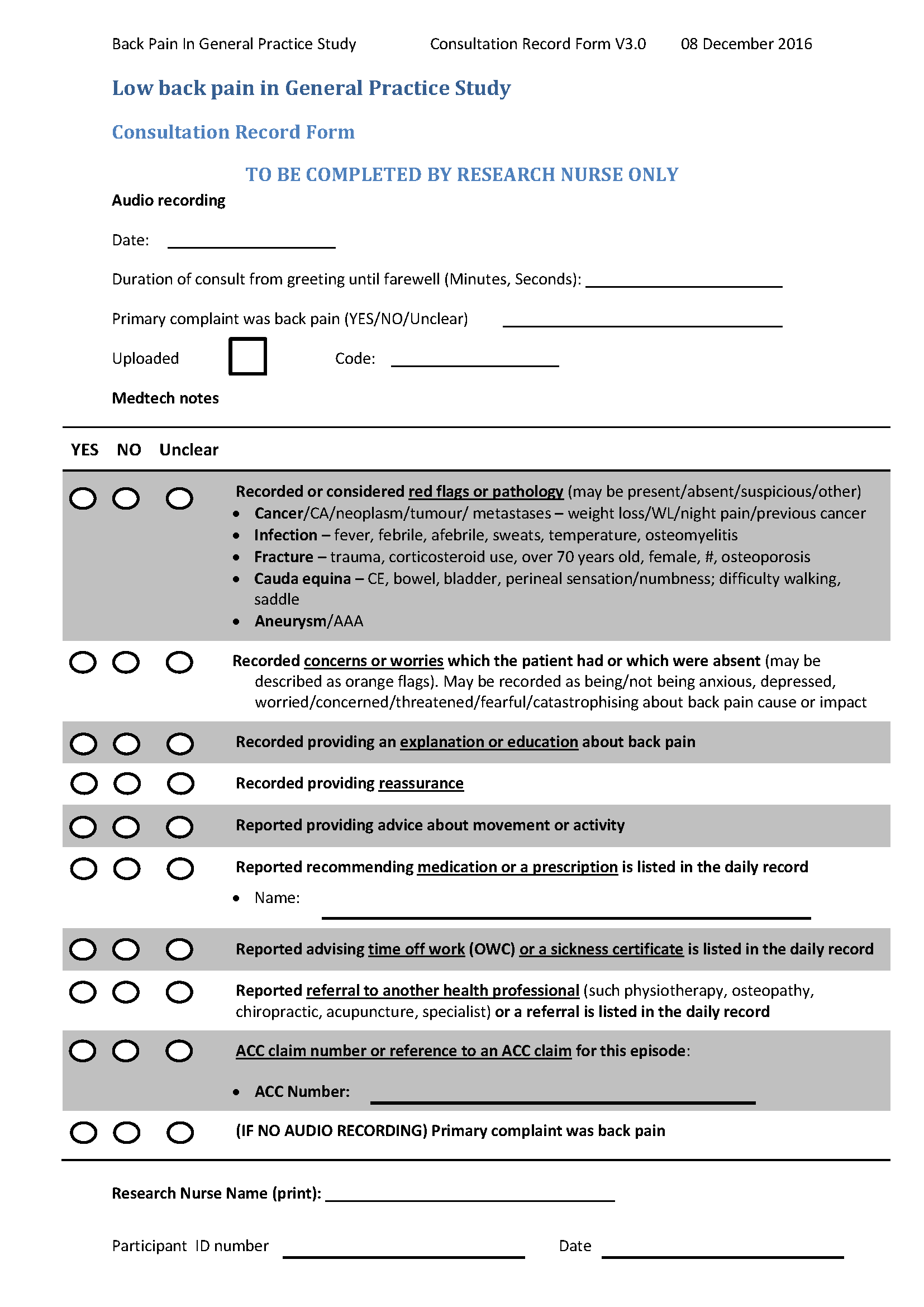


***Figure S5*. Consultation record form completed by the research nurse using data entered into the electronic medical record by the GP.**

| ***Table S15* Patient consultation outcomes/events as coded from Patient Management System notes**. | | | | | |
| --- | --- | --- | --- | --- | --- |
| **Recorded consultation component** | **Coding** | **FREE (n=126, 32 GPs)** | | **Control (n=100, 25 GPs)** | |
|  |  | **n** | **% with consultation element (95% CI)** | **n** | **% with consultation element (95% CI)** |
| Considered Red Flags | Yes | 53 | 42·4 (26·9 to 59·5) | 60 | 60·6 (44·2 to 74·9) |
|  | No | 70 | 56·0 (39·2 to 71·5) | 39 | 39·4 (25·1 to 55·8) |
|  | Unclear | 2 | 1·6 (0·4 to 6·4) | 0 | 0·0 (0·0 to 3·7)* |
| Considered patient concerns or worries | Yes | 42 | 33·6 (23·3 to 45.8) | 1 | 1·0 (0·1 to 7·7) |
|  | No | 81 | 64·8 (53·1 to 75·0) | 96 | 97·0 (90·2 to 99·1) |
|  | Unclear | 2 | 1·6 (0·4 to 6·6) | 2 | 2·0 (0·4 to 8·7) |
| Explained back pain | Yes | 75 | 60·0 (44·8 to 73·5) | 10 | 10·1 (4·2 to 22·4) |
|  | No | 47 | 37·6 (24·0 to 53·4) | 87 | 87·9 (75·7 to 94·4) |
|  | Unclear | 3 | 2·4 (0·7 to 7·5) | 2 | 2·0 (0·5 to 8·6) |
| Provided reassurance | Yes | 74 | 63·2 (45·9 to 77·7) | 5 | 5·6 (1·8 to 16·3) |
|  | No | 43 | 36·8 (22·3 to 54·1) | 84 | 94·4 (83·7 to 98·2) |
|  | Unclear | 0 | 0·0 (0·0 to 3·1)* | 0 | 0·0 (0·0 to 4·1)* |
| Provided advice about moving | Yes | 83 | 66·9 (53·7 to 77·9) | 22 | 22·2 (12·1 to 37·3) |
|  | No | 35 | 28·2 (17·6 to 42·0) | 77 | 77·8 (62·7 to 87·9) |
|  | Unclear | 6 | 4·8 (1·6 to 14·1) | 0 | 0·0 (0·0 to 0·0)* |
| Recommended medication/script | Yes | 103 | 82·4 (70·4 to 90·2) | 88 | 88·9 (81·8 to 93·5) |
|  | No | 21 | 16·8 (9·1 to 29·0) | 11 | 11·1 (6·5 to 18·2) |
|  | Unclear | 1 | 0·8 (0·1 to 6·0) | 0 | 0·0 (0·0 to 3·7) |
| Advised time off work or sickness certificate | Yes | 23 | 18·4 (12·1 to 26·9) | 24 | 24·2 (13·6 to 39·3) |
|  | No | 96 | 76·8 (70·2 to 82·3) | 74 | 74·7 (59·9 to 85·5) |
|  | Unclear | 6 | 4·8 (2·0 to 11·0) | 1 | 1·0 (0·1 to 7·9) |
| Referral to adjunct/specialist | Yes | 27 | 21·6 (14·5 to 30·9) | 49 | 49·5 (39·1 to 59·9) |
|  | No | 93 | 74·4 (63·8 to 82·7) | 49 | 49·5 (38·7 to 60·3) |
|  | Unclear | 5 | 4·0 (1·0 to 15·2) | 1 | 1·0 (0·1 to 7·9) |
| Linked consultation to an ACC claim | Yes | 68 | 54·4 (43·4 to 64·9) | 67 | 69·1 (59·3 to 77·4) |
| (new or existing) | No | 54 | 43·2 (32·6 to 54·4) | 30 | 30·9 (22·6 to 40·7) |
|  | Unclear | 3 | 2·4 (0·8 to 7·3) | 0 | 0·0 (0·0 to 3·7)* |
| *for response options with no respondents, confidence intervals were calculated using the binomial distribution (not accounting for clustering). ACC, Accident Compensation Corporation. | | | | | |

### Consultation audio recording and treatment fidelity assessment

#### Methods

Written informed consent was sought from GPs and patient participants to allow audio-recording of the recruitment consultation. Recording only occurred when both the GP and the patient participant provided this consent. The consultation was recorded by the research nurse who placed a digital audio recorded in the consultation room at the start of the consultation and retrieved this at the end of the consultation. The research nurse did not remain in the consultation room while the consult was occurring.

The research nurse who obtained each recording listened to the recording to a) confirm that back pain was the main reason for consultation and b) record the duration of the consultation (from the greeting to the farewell).

One audio-recording was randomly selected for each GP who had consented to audio-recording and treated at least one patient participant who had also consented to recording. Treatment fidelity was analysed in these recordings using a structured checklist containing multiple consultation behaviour items. Subsets of these behaviours were pre-determined as i) integral elements of FREE (4 out of 5 elements required to classify a consultation as FREE concordant i.e. fidelity to intervention delivery) and ii) representing 15 key elements of FREE (five from each of the three domains of FREE). The researcher analysing the recordings was blind to group allocation and to the arrangement of assessed elements in these subsets. This experienced health researcher was previously unexposed to the intervention and was trained in identification of these elements. This researcher and a member of the research team who was also blind to group allocation (TD) undertook pilot analysis of six audio recordings that were not part of the analysis set. Analysis results were compared and discrepancies were resolved by consensus with the assistance of the primary investigator. This process resulted in some refinement of the structured checklist to aid clarity and consistency, prior to analysis of the randomly selected set.

Separate analyses were conducted for each item. Firstly, each selected consultation was analysed for the number of integral elements (0 to 5) and whether it included at least four elements (binary outcome); followed by the number of key elements identified in each of the three FREE domains. Analyses report the proportion of consultations in each of these classifications by study arm. As only one audio recording was audited per consenting GP, analysis did not need to account for clustering by GP in the estimation of the confidence intervals.

#### Results

Audio-recordings were obtained for 60 intervention and 78 control consultations. Of the 32 FREE arm GPs who saw a patient in the study, there was at least one audio recorded consultation for 23 GPs; 7 GPs in the FREE arm declined to be recorded and 2 consenting GPs were unable to be recorded because their study patients declined. Of the 25 control arm GPs who saw a patient in the study, there was at least one audio recorded consultation for 24 GPs; 1 consenting GP was unable to be analysed as the audio recording cut out 5 minutes into the consultation. Five GP participants did not have any recruited patients in the study.

Mean consultation duration of recorded consultations in the FREE arm was 22·8 minutes (SD = 8·5; 95% CI 19·1 to 26·5) and in the Control arm was 16·5 minutes (SD = 4·1; 14·7 to 18·2). The mean duration was significantly longer in the FREE arm (mean difference = 6·3 minutes longer, 2·3 to 10·4).

The majority of GPs consultations in the FREE arm met the pre-defined ‘FREE concordant’ threshold of at least 4 of 5 criteria (82·6%, 61·2 to 95·0) while no GPs in the control arm had such a consultation (0·0%, 0·0 to 11·7) (Table S15). GPs in the FREE arm also included a higher number of FREE elements in these consultations compared to the Control arm GPs (Table S16).

| ***Table S16.* Coded results of audio recordings regarding concordance of consultation with FREE principles (one recording analysed per consenting GP).** | | | | | |
| --- | --- | --- | --- | --- | --- |
| **Criteria used** | | **FREE (n=23, 4 practices)** | | **Control (n=24, 4 practices)** | |
|  |  | **n** | **% (95% CI)** | **n** | **% (95% CI)** |
| **FREE concordance** |  |  |  |  |  |
| Number of integral | 0 | 0 | 0·0 (0·0 to 12·2) | 5 | 20·8 (7·1 to 42·2) |
| elements included | 1 | 2 | 8·7 (1·1 to 28·0) | 9 | 37·5 (18·8 to 59·4) |
|  | 2 | 1 | 4·3 (0·1 to 21·9) | 5 | 20·8 (7·1 to 42·2) |
|  | 3 | 1 | 4·3 (0·1 to 21·9) | 5 | 20·8 (7·1 to 42·2) |
|  | 4 | 8 | 34·8 (16·4 to 57·3) | 0 | 0·0 (0·0 to 11·7) |
|  | 5 | 11 | 47·8 (26·8 to 69·4) | 0 | 0·0 (0·0 to 11·7) |
| Meets ≥ 4 criteria | No | 4 | 17·4 (5·0 to 38·8) | 24 | 100·0 (88·3 to 100·0) |
|  | Yes | 19 | 82·6 (61·2 to 95·0) | 0 | 0·0 (0·0 to 11·7) |
| **FREE performance items** |  |  |  |  |  |
| # Explore domain items | 0 | 1 | 4·3 (0·1 to 21·9) | 15 | 62·5 (40·6 to 81·2) |
| included | 1 | 2 | 8·7 (1·1 to 28·0) | 9 | 37·5 (18·8 to 59·4) |
|  | 2 | 7 | 30·4 (13·2 to 52·9) | 0 | 0·0 (0·0 to 11·7) |
|  | 3 | 7 | 30·4 (13·2 to 52·9) | 0 | 0·0 (0·0 to 11·7) |
|  | 4 | 3 | 13·0 (2·8 to 33·6) | 0 | 0·0 (0·0 to 11·7) |
|  | 5 | 3 | 13·0 (2·8 to 33·6) | 0 | 0·0 (0·0 to 11·7) |
| # Explain domain items | 0 | 3 | 13·0 (2·8 to 33·6) | 10 | 41·7 (22·1 to 63·4) |
| included | 1 | 3 | 13·0 (2·8 to 33·6) | 10 | 41·7 (22·1 to 63·4) |
|  | 2 | 2 | 8·7 (1·1 to 28·0) | 2 | 8·3 (1·0 to 27·0) |
|  | 3 | 5 | 21·7 (7·5 to 43·7) | 2 | 8·3 (1·0 to 27·0) |
|  | 4 | 8 | 34·8 (16·4 to 57·3) | 0 | 0·0 (0·0 to 11·7) |
|  | 5 | 2 | 8·7 (1·1 to 28·0) | 0 | 0·0 (0·0 to 11·7) |
| # Experiment domain items | 0 | 2 | 8·7 (1·1 to 28·0) | 14 | 58·3 (36·6 to 77·9) |
| included | 1 | 2 | 8·7 (1·1 to 28·0) | 7 | 29·2 (12·6 to 51·1) |
|  | 2 | 5 | 21·7 (7·5 to 43·7) | 3 | 12·5 (2·7 to 32·4) |
|  | 3 | 2 | 8·7 (1·1 to 28·0) | 0 | 0·0 (0·0 to 11·7) |
|  | 4 | 5 | 21·7 (7·5 to 43·7) | 0 | 0·0 (0·0 to 11·7) |
|  | 5 | 7 | 30·4 (13·2 to 52·9) | 0 | 0·0 (0·0 to 11·7) |

·

### GP reported clinical management of vignette case

#### Methods

All GP participants were asked to indicate the management advice they would provide for a case described in a clinical vignette. ^80^ Data were collected at baseline, 4 weeks and 4 months (Table S17).

Reported clinical behaviour relating to the case vignette (categorical outcome from five options) were compared in two ways^80^:

- - 1. changes in response proportions of reported behaviour across the entire range of responses will be compared between arms using a generalised linear mixed model with a cumulative logit link function (to allow for ordinal nature of outcome), adjusted for baseline values and including a random effect for GP practice (to handle clustering)
    2. changes in the proportions of consultations following 'guideline-consistent' reported behaviour (in contrast to 'guideline-inconsistent' behaviour) will be compared between arms using a generalised linear mixed model with a logit link (i.e. akin to a logistic regression specification), with responses categorised into two levels (“guideline consistent” vs “guideline inconsistent”). This analysis will be adjusted for baseline values and include a random effect for GP practice to handle clustering

| ***Table S17.* GP reported management of a vignette case.** | |
| --- | --- |
| **GP management item** | **Data collection time points** |
| Clinical case vignettes of patients with non-specific LBP with items relating to advice given to the patient about work, activity and rest   - Categorical outcome with five response options | 0, 4w, 4m |

#### Results

GPs in the FREE arm were more likely to give guideline-consistent activity vignette recommendations at four weeks (unadjusted estimates: 87·9% vs 62·1% for FREE and Control respectively; OR = 5·8, 95% CI 1·3 to 25·9) though this difference was smaller at 4 months (unadjusted estimates: 87·9% vs 69·0% for FREE and Control respectively; OR = 4·6, 95% CI 0·7 to 30·5) (S18). Neither the Work nor Rest vignettes showed substantial differences by study arm in terms of numbers of respondents reporting guideline-consistent management (Table S18).

Analysis of the vignette responses as ordinal measures (Table S19 shows the original 1-5 scaling in the response options) strongly suggested that GPs in the FREE arm had moved to a higher level of consistency with guideline recommendations for activity and work at 4 weeks and 4 months (for example, at Week 4 the majority of FREE GPs in the “consistent” zone for the Work vignette were in either level 4 or level 5; while the majority of Control GPs in the consistent zone were in the middle response category (level 3). These interpretations were generally supported by cumulative logistic regression models that indicated statistically significant ordinal effects comparing the FREE and Control arms (accounting for clustering of GPs). Ordinal trends were less strong in relation to rest recommendations but FREE arm GPs were still more likely to recommend avoiding bed rest entirely than control arm GPs at 4 months.

| ***Table S18*. Descriptive statistics (frequency, %) for GP vignette responses categorised with a binary outcomes as ‘guideline consistent’ or ‘guideline inconsistent’ at baseline and follow-up; and p-values comparing responses at follow-up by study arm**. | | | | | | | | | | | |
| --- | --- | --- | --- | --- | --- | --- | --- | --- | --- | --- | --- |
| **Vignette item** |  | **Baseline** | |  | **4 weeks (post-training)** | | |  | **4 months (post-training)** | | |
|  |  | **FREE** | **Control** |  | **FREE** | **Control** | **p-value*** |  | **FREE** | **Control** | **p-value*** |
|  |  | **(n=33†,  4 practices)** | **(n=29,  4 practices)** |  | **(n=33,  4 practices)** | **(n=29,  4 practices)** |  |  | **(n=33,  4 practices)** | **(n=29,  4 practices)** |  |
|  |  | **n (%)** | **n (%)** |  | **n (%)** | **n (%)** |  |  | **n (%)** | **n (%)** |  |
| #1 (Activity) | Not consistent | 11 (33·3) | 11 (37·9) |  | 4 (12·1) | 11 (37·9) | 0·030 |  | 4 (12·1) | 9 (31·0) | 0·083 |
|  | Consistent | 22 (66·7) | 18 (62·1) |  | 29 (87·9) | 18 (62·1) |  |  | 29 (87·9) | 20 (69·0) |  |
| #2 (Work) | Not consistent | 10 (30·3) | 8 (27·6) |  | 2 (6·1) | 4 (13·8) | 0·376 |  | 4 (12·1) | 4 (13·8) | 0·926 |
|  | Consistent | 23 (69·7) | 21 (72·4) |  | 31 (93·9) | 25 (86·2) |  |  | 29 (87·9) | 25 (86·2) |  |
| #3 (Rest) | Not consistent | 4 (12·1) | 6 (20·7) |  | 0 (0·0) | 2 (6·9) | Non-est**‡** |  | 1 (3·0) | 1 (3·4) | 0·951 |
|  | Consistent | 29 (87·9) | 23 (79·3) |  | 33 (100·0) | 27 (93·1) |  |  | 32 (97·0) | 28 (96·6) |  |
| * p-value for hypothesis test for odds of having vignette response consistent with best practice in FREE arm relative to Control arm. Hypothesis test adjusted for baseline vignette response for that item and practice size. † Baseline value for one GP with baseline-only data excluded here (see GP baseline scores table for description of full sample at baseline.) **‡** Odds ratio could not be estimated here given 100% consistency with guideline in the FREE arm. | | | | | | | | | | | |

| ***Table S19.* Descriptive statistics (frequency, %) for full detail of GP vignette responses at baseline and follow-up; and odds ratios comparing responses at follow-up by study arm (using an ordinal model).** | | | | | | | | | | | |
| --- | --- | --- | --- | --- | --- | --- | --- | --- | --- | --- | --- |
| **Vignette item** |  | **Baseline** | |  | **4 weeks (post-training)** | | |  | **4 months (post-training)** | | |
|  |  | **FREE** | **Control** |  | **FREE** | **Control** | **Ordinal p-value*** |  | **FREE** | **Control** | **Ordinal p-value*** |
|  |  | **(n=33†,**  **4 practices)** | **(n=29,**  **4 practices)** |  | **(n=33†,**  **4 practices)** | **(n=29,**  **4 practices)** |  |  | **(n=33†,**  **4 practices)** | **(n=29,**  **4 practices)** |  |
|  | **Level** | **n (%)** | **n (%)** |  | **n (%)** | **n (%)** |  |  | **n (%)** | **n (%)** |  |
| #1 (Activity) | 1 | 0 (0·0) | 0 (0·0) |  | 0 (0·0) | 0 (0·0) |  |  | 0 (0·0) | 0 (0·0) |  |
|  | 2 | 7 (21·2) | 4 (13·8) |  | 0 (0·0) | 6 (20·7) |  |  | 2 (6·1) | 4 (13·8) |  |
|  | 3 | 4 (12·1) | 6 (20·7) |  | 4 (12·1) | 4 (13·8) | <0·001 |  | 1 (3·0) | 5 (17·2) | <0·001 |
|  | 4 | 17 (51·5) | 13 (44·8) |  | 8 (24·2) | 13 (44·8) |  |  | 7 (21·2) | 14 (48·3) |  |
|  | 5 | 5 (15·2) | 5 (17·2) |  | 21 (63·6) | 5 (17·2) |  |  | 22 (66·7) | 6 (20·7) |  |
| #2 (Work) | 1 | 0 (0·0) | 0 (0·0) |  | 0 (0·0) | 0 (0·0) |  |  | 0 (0·0) | 0 (0·0) |  |
|  | 2 | 10 (30·3) | 6 (20·7) |  | 2 (6·1) | 4 (13·8) |  |  | 3 (9·1) | 4 (13·8) |  |
|  | 3 | 18 (54·5) | 16 (55·2) |  | 10 (30·3) | 21 (72·4) | <0·001 |  | 8 (24·2) | 18 (62·1) | <0·001 |
|  | 4 | 0 (0·0) | 1 (3·4) |  | 8 (24·2) | 2 (6·9) |  |  | 4 (12·1) | 2 (6·9) |  |
|  | 5 | 5 (15·2) | 4 (13·8) |  | 13 (39·4) | 2 (6·9) |  |  | 17 (51·5) | 5 (17·2) |  |
| #3 (Rest) | 1 | 0 (0·0) | 0 (0·0) |  | 0 (0·0) | 0 (0·0) |  |  | 0 (0·0) | 0 (0·0) |  |
|  | 2 | 0 (0·0) | 0 (0·0) |  | 0 (0·0) | 0 (0·0) |  |  | 0 (0·0) | 0 (0·0) |  |
|  | 3 | 3 (9·1) | 5 (17·2) |  | 0 (0·0) | 2 (6·9) | 0·080 |  | 0 (0·0) | 1 (3·4) | 0·039 |
|  | 4 | 15 (45·5) | 19 (65·5) |  | 11 (33·3) | 18 (62·1) |  |  | 9 (27·3) | 20 (69·0) |  |
|  | 5 | 14 (42·4) | 4 (13·8) |  | 22 (66·7) | 9 (31·0) |  |  | 23 (69·7) | 8 (27·6) |  |
| Higher scores represent increased guideline consistency. * p-value for hypothesis test for having a higher level of vignette response consistent with best practice in FREE arm relative to Control arm (as an ordinal outcome). Ordinal logistic regression adjusted for baseline vignette response for that item. † Baseline value for one GP with baseline-only data excluded here (see GP baseline scores table for description of full sample at baseline.) | | | | | | | | | | | |

## Economic analyses

### Healthcare utilisation

#### Method

Health economic data and some secondary patient outcomes (number of days off work/on restricted duties, and quantities of medications taken) were self-reported by patients through the Otago Costs and Consequences Questionnaire^24^ for LBP (OCC-Q-LBP) as completed by patients at 2 weeks, 3 months and 6 months.

Cost-utility analysis estimated mean incremental cost per quality-adjusted life year (QALY) and monetary incremental net benefit (INB) gained from health care system and societal perspectives, and from the Accident Compensation Corporation (ACC; no fault compulsory insurance cover for all personal injury in New Zealand) perspective. Reference costs were assigned for i) all health care items, to allow direct comparison and decrease patient recall requirements; ii) paid work (based on gender- and age-specific mean income); and iii) unpaid/voluntary work (based upon the minimum wage).

Multiple imputation was used to address missing data in the cost and health utility outcomes measures. Patterns of missing data and the correlations between variables were examined to determine appropriate imputation models for each variable.^25^ Imputations were created using predictive mean matching (continuous variables) and logistic regression models (dichotomous variables). As cost data were highly skewed, a large number of imputed datasets (m=50) were created to ensure cost estimates would not be driven solely by a small number of large imputed values in one treatment arm. All multiple imputation analyses were conducted using mice (version 2.46.0)^26^ in *R* version 3.5.0.

Comparison of days off work and days on restricted duties was conducted using the multiply-imputed versions of these two variables as these measures were accumulated over the entire follow-up period (and hence mixed models could not account for missing data as there were no interim data). Adjusted linear mixed models (allowing for clustering and adjusted for standard factors as above) were used to analyse these two outcomes for each of the 50 imputed datasets. The resulting estimates were recombined following the standard rules for pooling results across imputed datasets.^27^

#### Results

There were few significant differences between the intervention and control groups in healthcare utilisation over the 6-month follow-up period. As is typical for such data, wide variability among the data resulted in wide confidence intervals, with most differences inconclusive (Table S20). Strong differences that emerged included patients in the FREE arm were less likely to see a physiotherapist for their back pain (OR = 0·4, 95% CI 0·2 to 0·7; mean difference in number of visits = –1·7, 95% CI –3·0 to –0·4), and less likely to have a linked ACC claim (OR = 0·5, 95% CI 0·3 to 0·8).

Mean treatment costs over 6 months were $665 (health system perspective), $114 (ACC perspective), and $2387 (societal perspective) in the FREE arm, and $997, $608, and $3069, respectively, in the control group (Table S21). These group differences were not statistically significant from the societal, health system, or ACC perspectives. The confidence interval for the cost difference from the ACC perspective indicated a high likelihood of cost savings ($494; 95% CI from $991 saving with FREE to a $2 extra cost; p=0·051).

| ***Table S20*. Average quantity of healthcare used and percentage of patients reporting any use (95% CI).** | | | | |
| --- | --- | --- | --- | --- |
| **Healthcare type** | **Quantity of healthcare used**  **Mean (95% CI)** | | **Percentage using any healthcare**  **Percent (95% CI)** | |
|  | **FREE  (n = 126; 32 GPs)** | **Control  (n = 100; 25 GPs)** | **FREE  (n = 126; 32 GPs)** | **Control  (n = 100; 26 GPs)** |
| **Visits to Doctors** | | | | |
| GP (other than initial visit) | 1·0 (0·6, 1·4) | 1·0 (0·5, 1·5) | 32·8 (24·8, 40·4) | 33·4 (24·4, 43·8) |
| Other doctors | 0·3 (0·1, 0·4) | 0·3 (0·1, 0·5) | 11·6 (6·4, 20·1) | 11·1 (5·7, 20·6) |
| **Visits to Other Health Professionals** | | | | |
| Physiotherapist | 1·2 (0·6, 1·9) | 2·9 (1·7, 4·2) | 22·5 (15·7, 31·1) | 41·7 (32·0, 52·0) |
| Chiropractor | 0·8 (0·2, 1·3) | 0·6 (0·1, 1·1) | 11·3 (6·5, 19·1) | 10·3 (5·3, 19·1) |
| Massage therapist | 0·3 (0·0, 0·6) | 0·5 (0·1, 0·8) | 6·9 (3·2, 14·5) | 11·3 (6·1, 20·1) |
| Other health professionals† | 0·8 (0·2, 1·3) | 1·0 (0·1, 1·8) | 18·9 (12·5, 27·5) | 16·7 (9·8, 27·0) |
| **Investigations, Tests, Injections, or Procedures** | | | | |
| Blood tests | 0·2 (0·1, 0·4) | 0·2 (0·0, 0·4) | 14·9 (9·3, 22·9) | 9·1 (4·3, 18·3) |
| X-rays | 0·2 (0·1, 0·3) | 0·3 (0·2, 0·4) | 12·9 (7·6, 21·1) | 22·0 (14·6, 31·7) |
| Other procedures | 0·3 (0·1, 0·5) | 0·5 (0·2, 0·8) | 13·5 (8·0, 21·8) | 15·2 (8·8, 24·8) |
| **Hospital Services** | | | | |
| Any hospital services (ambulance, A&E, admissions) | 0·1 (0·0, 0·3) | 0·1 (0·0, 0·2) | 5·6 (2·3, 13·1) | 5·1 (1·8, 13·7) |
| **Pain Medications (total units (e·g· pills) over 6 months)** | | | | |
| Paracetamol | 228·6 (161·7, 295·6) | 206·7 (141·9, 271·5) | 61·9 (52·7, 70·3) | 58·8 (48·3, 68·5) |
| Codeine | 25·1 (2·5, 47·8) | 24·9 (6·6, 43·2) | 14·3 (8·9, 22·3) | 17·1 (10·6, 26·4) |
| Paracetamol & codeine | 23·7 (9·0, 38·3) | 35·9 (12·0, 59·7) | 17·1 (11·0, 25·5) | 19·2 (12·3, 28·7) |
| NSAIDs | 118·6 (74·6, 162·6) | 115·1 (75·3, 154·9) | 61·1 (51·8, 69·6) | 62·7 (52·0, 72·3) |
| Anti-inflammatory cream/ gels | 43·0 (2·3, 83·7) | 23·3 (2·4, 44·3) | 27·1 (19·6, 36·3) | 21·6 (14·1, 31·6) |
| Other pain medications | 67·3 (30·3, 104·4) | 51·1 (19·7, 82·4) | 27·7 (20·0, 37·0) | 29·5 (20·9, 39·9) |
| **Other Medications (total units (e·g· pills) over 6 months)** | | | | |
| Stomach irritation | 44·8 (22·1, 67·6) | 28·1 (7·2, 49·1) | 23·2 (16·3, 32·1) | 17·8 (11·1, 27·4) |
| Mood or sleep | 43·8 (17·6, 70·1) | 36·7 (17·7, 55·7) | 20·1 (13·6, 28·5) | 24·8 (17·0, 34·6) |
| Other meds or supplements | 43·1 (4·2, 81·9) | 64·7 (13·7, 115·7) | 13·8 (8·5, 21·7) | 15·9 (9·5, 25·4) |
| **Services in the Home** | | | | |
| Any home services | 0·5 (0·0, 1·1) | 0·9 (0·1, 1·6) | 8·9 (4·6, 16·5) | 9·2 (4·2, 18·9) |
| **Aids and Adaptations** | | | | |
| Back support for sitting | – | – | 11·5 (6·7, 19·1) | 12·6 (7·0, 21·4) |
| Back brace | – | – | 11·1 (6·4, 18·6) | 4·4 (1·4, 12·8) |
| Other aids or adaptations | – | – | 15·1 (9·4, 23·2) | 13·9 (7·9, 23·5) |
| **Impact on Work** | | | | |
| Took time off work | 4·8 (2·4, 7·3) | 5·6 (2·6, 8·7) | 45·2 (36·3, 54·4) | 52·3 (41·9, 62·5) |
| Worked fewer hours or restricted duties | 3·0 (1·2, 4·8) | 4·4 (1·2, 7·6) | 35·4 (27·2, 44·5) | 37·2 (27·8, 47·7) |
| **ACC Claims** | | | | |
| Linked ACC claim | – | – | 33·3 (25·6, 42·1) | 51·0 (41·2, 60·8) |
| GP, general practitioner; NSAIDs, non-steroidal inti-inflammatory drugs; ACC, Accident Compensation Corporation.  † Category includes professionals with less than 10% utilisation in either arm (osteopath, acupuncturist, nurse, occupational therapist, complimentary and alternative therapist). | | | | |

| ***Table S21.* Group mean outcomes over 6-month follow-up (95% CI)·** | | | | |
| --- | --- | --- | --- | --- |
|  | **FREE (n = 126; 32 GPs)** | **Control (n = 100; 25 GPs)** | **Difference** | **p-value** |
| QALYs | 0·340 (0·325, 0·354) | 0·331 (0·314, 0·348) | 0·009 (-0·014, 0·031) | 0·434 |
| Total Costs ($) | 2387 (1334, 3433) | 3069 (1891, 4247) | -682 (-2258, 894) | 0·395 |
| Health System Costs ($) | 665 (330, 999) | 997 (642, 3424) | -332 (-820, 156) | 0·181 |
| ACC Costs ($) | 114 (-216, 444) | 608 (238, 3439) | -494 (-991, 2) | 0·051 |
| ACC, Accident Compensation Corporation; QALY, quality adjusted life year.· | | | | |

### Cost-effectiveness evaluation

#### Method

Incremental cost-effectiveness ratio (ICER) and the monetary incremental net benefit (INB) with 95% confidence intervals around the estimates were calculated. Incremental cost and effect data are displayed on a cost-effectiveness plane, and cost-effectiveness acceptability curves (CEAC) were calculated to determine the likelihood that FREE was considered cost-effective using one, two, and three times GDP per capita as policy-relevant willingness-to-pay (WTP) thresholds (2017 NZ$).

#### Results

Patients in the intervention group experienced an average of 0·34 QALYs (out of a possible 0.5) over the 6-month follow-up period, compared with 0·33 QALYs in the control group (Table S21). After adjusting for baseline covariates, treatment effects were estimated to be not clinically significant at 0·005 QALYs gained in the intervention group (95% CI: –0·014 to 0·024), with cost savings (per patient) of $270 (95% CI: –$192 to $732) from the health system perspective, $422 (95% CI: –$50 to $895) from the ACC perspective, and $337 (95% CI: –$1168 to $1842) from the societal perspective.

As the intervention dominated the control treatment (with higher QALYs and lower costs), albeit with wide uncertainty intervals, incremental cost-effectiveness ratios are not an appropriate statistic to summarise the cost-effectiveness findings. As pre-specified in the protocol,^2^ we therefore present incremental net benefits (INBs), at willingness-to-pay thresholds of 1× GDP/capita ($55,615), 2× GDP/capita ($111,230), and 3× GDP/capita ($166,845) per QALY gained, with 95% confidence intervals. The intervention had a positive INB at all willingness-to-pay thresholds, from all three cost perspectives, but did not reach statistical significance in any scenario (Table S22).

| ***Table S22*.** **Cost-effectiveness of intervention relative to control treatment (95% CI).** | | | |
| --- | --- | --- | --- |
| **Economic measure** | **Societal Perspective** | **Health System Perspective** | **ACC Perspective** |
| ICER ($ / QALY gained) | –69002* | –55223* | –86440* |
| *INB ($)* |  |  |  |
| 1x GDP/capita ($55,615) | 609 (–1385, 2603) | 542 (–678, 1762) | 695 (–496, 1885) |
| 2x GDP/capita ($111,230) | 881 (–1937, 3699) | 814 (–1419, 3046) | 966 (–1231, 3164) |
| 3x GDP/capita ($166,845) | 1153 (–2604, 4911) | 1086 (–2184, 4356) | 1238 (–1995, 4472) |
| ACC, Accident Compensation Corporation; ICER, incremental cost effectiveness ratio; QALY, quality adjusted life year; INB, incremental net benefit; GDP, gross domestic product. * Intervention dominates control (higher QALYs and lower costs). | | | |

To evaluate uncertainty in the estimated cost-effectiveness outcomes, we generated confidence ellipses for the QALY gains and cost savings, for each of the cost perspectives specified in the analysis, on the cost-effectiveness plane (incremental costs vs. incremental QALYs), and cost-effectiveness acceptability curves, showing the probability that each intervention would be considered cost-effective at the three specified willingness-to-pay thresholds.

The intervention had greater than 50% probability of being cost-effective at all willingness-to-pay levels, with a probability of cost-effectiveness of between approximately 70% and 85%, from all three cost perspectives, at policy-relevant willingness-to-pay thresholds of 1 to 3 times GDP/capita (Figures S6 and S7).


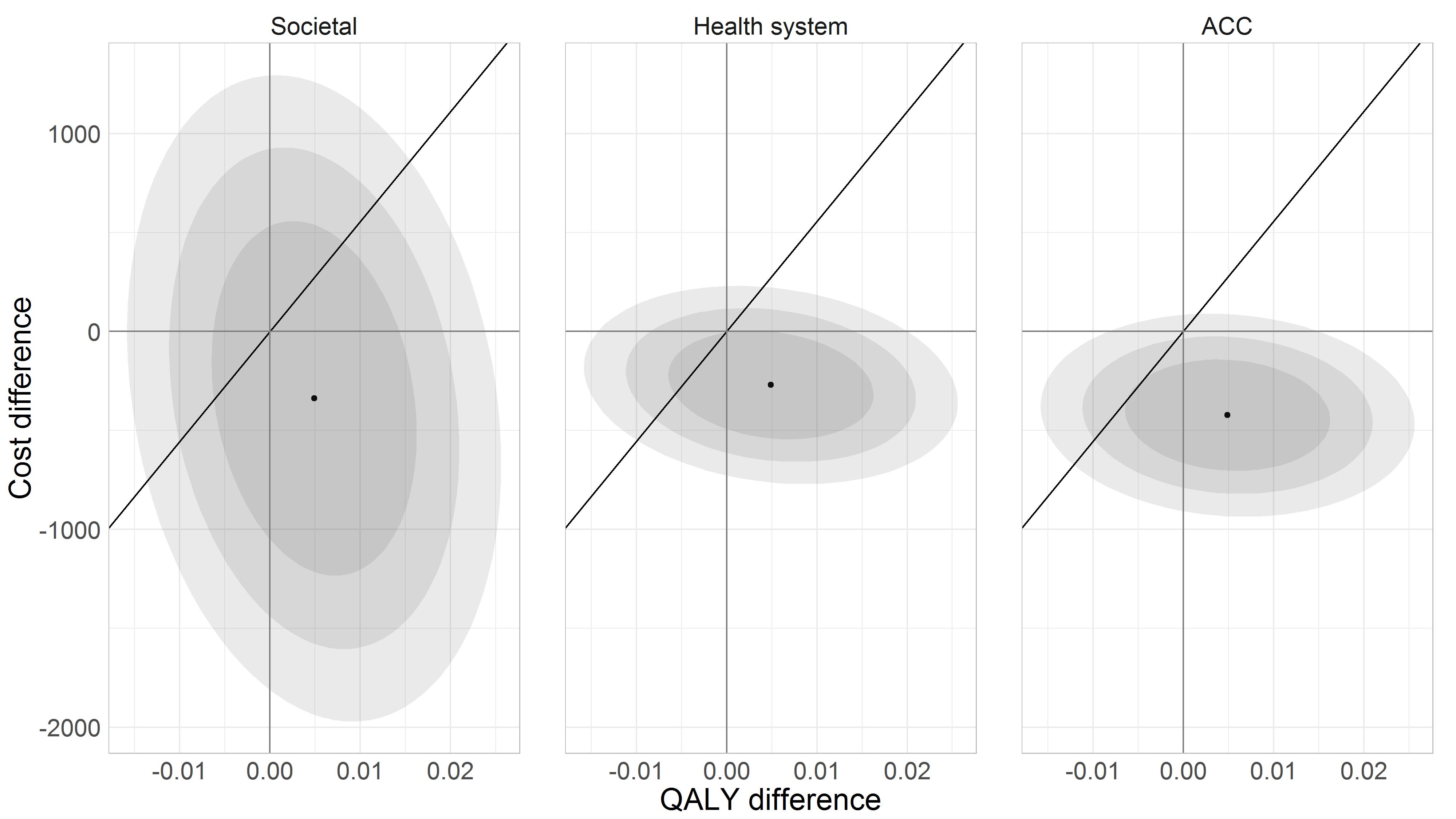


***Figure S6*.** **Cost-effectiveness plane.** Shaded areas show 50% (darker), 75%, and 90% (lighter) confidence ellipses. Solid line shows willingness to pay threshold at 1× GDP per capita; areas below and to the right of the line indicate the intervention is cost-effective relative to control, above and to the left indicate the intervention is not cost-effective. ACC, Accident Compensation Corporation; QALY, quality adjusted life year.


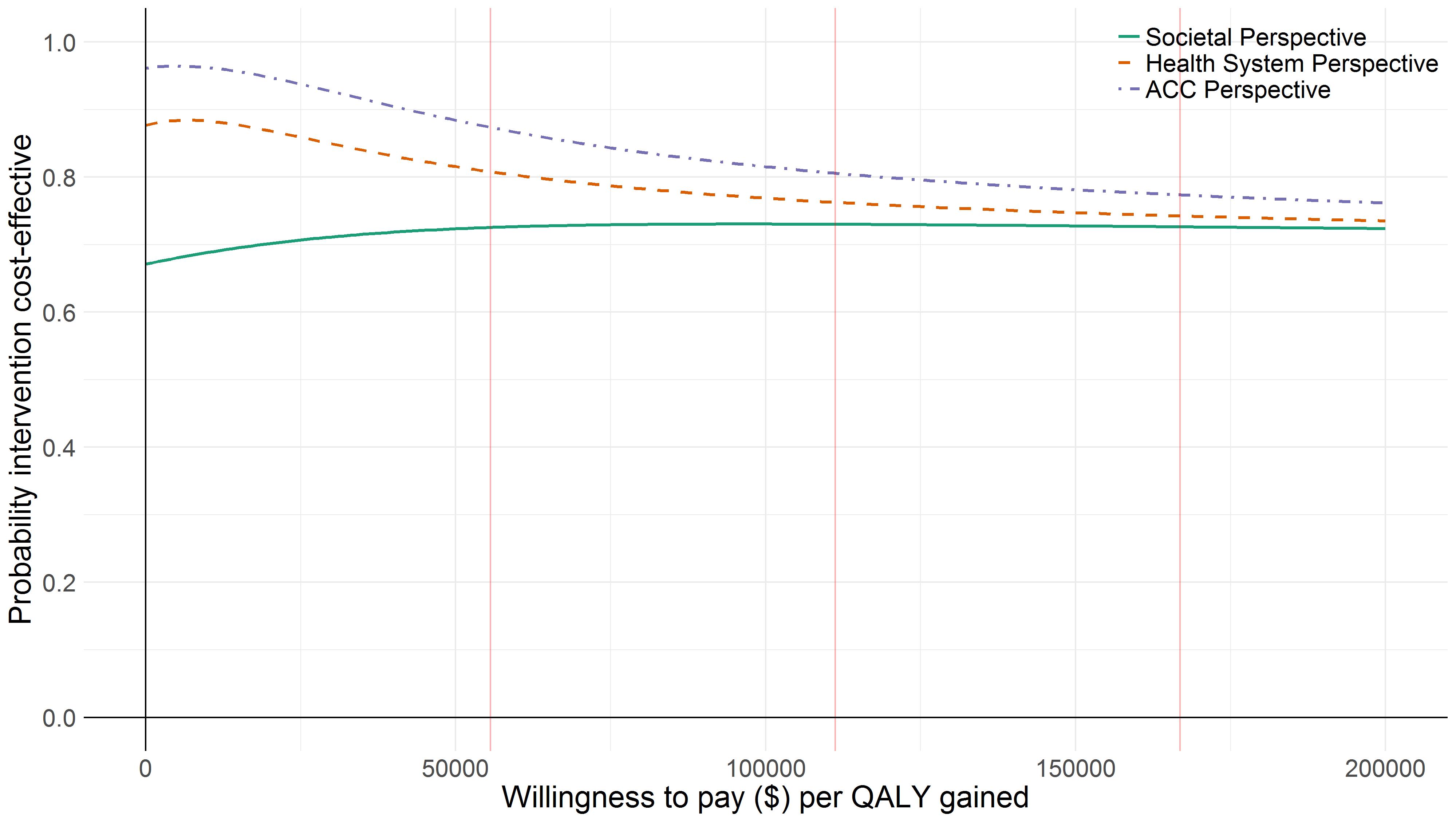


***Figure S7*. Cost-effectiveness acceptability curves.** Vertical lines indicate willingness to pay thresholds at 1×, 2×, and 3× GDP per capita, respectively. ACC, Accident Compensation Corporation; QALY, quality adjusted life year.

### Sensitivity analyses

Similar results were found across several sensitivity analyses. Restricting the sample to those participants meeting the original recruitment criteria (aged under 65, experiencing back pain for less than 6 weeks and not having received other treatment for back pain in the past 3 months) resulted in incremental net benefits, at the intermediate 2× GDP/capita level, of $892 (health system perspective), $1081 (ACC perspective), and $344 (societal perspective), with probabilities of being cost-effective of 74%, 74%, and 73%, respectively.

Using robust regression methods, which put less weight on outlying observations to reduce the impact of chance variability introduced by large costs in only a small number of individuals, estimated incremental net benefits were smaller (but more precisely estimated), at $262 (health system perspective), $175 (ACC perspective), and $284 (societal perspective), and probabilities of being cost-effective were 59%, 56%, and 60%, respectively.

A sensitivity analysis was performed for participants who received an ACC claim because, from the ACC perspective, costs were influenced by differing proportions of participants with ACC claims in each group.

At recruitment, 131 participants (64/126) FREE; 67/100 control) had an ACC claim recorded as part of their GP consultation. At the six month survey point 106/131 participants (51 FREE; 55 control) gave consent to communicate their name and claim number to ACC for data extraction.

At the six month survey point an additional 92 participants (FREE 60; control 32) were asked whether they had an ACC number (i.e a claim that was not noted at time of initial consult or received subsequently). Of these 92 participants, 12 (FREE 10; control 2) reported they had an ACC claim and 10 (FREE 9; control 1) gave consent to communicate their name and number to ACC for data extraction. This provided a total of 61 FREE arm and 57 control arm participants who considered they had a relevant ACC claim and consented to ACC data extraction. These participants were matched with ACC data using ACC claim numbers, demographic details and National Health Index numbers. No matching ACC claim was identified for 21 (13 FREE, 8 control) participants, resulting in ACC data being extracted for 101 (49 FREE; 52 control) participants. Of these, six claims (5 FREE; 1 control) had zero costs recorded over the 6-month follow-up period and two (both FREE) had claims that were not related to LBP (one knee injury and one elbow injury), consequently, a total of 93 participants (42 FREE; 51 control) with ACC costs related to LBP were entered in the analysis.

Stronger QALY gains and treatment cost-effectiveness were found for the subgroup of patients for whom an ACC claim was accepted for their back pain. Adjusted QALY gains were 0·033 for these patients (95% CI 0·003 to 0·063). Cost savings were also larger in this subgroup, at $544 (95% CI –143 to 1231) from the health system perspective, $704 (95% CI –363 to 1772) from the ACC perspective, and $1946 (95% CI –352 to 4244) from the societal perspective, although due to wide uncertainty, partly as a result of the smaller sample size, these did not quite reach statistical significance. Incremental net benefits were significantly greater than zero, from all cost perspectives and at all three willingness to pay levels considered (Table S23).

| ***Table S23.* Cost-effectiveness of intervention relative to control treatment (95% CI), patients with ACC claims.** | | | |
| --- | --- | --- | --- |
| **Economic measure** | **Societal Perspective** | **Health System Perspective** | **ACC Perspective** |
| ICER ($ / QALY gained) | –59200* | –16552* | –21425* |
| *INB ($)* |  |  |  |
| 1x GDP/capita ($55,615) | 3774 (712, 6836) | 2372 (481, 4263) | 2532 (459, 4606) |
| 2x GDP/capita ($111,230) | 5601 (1230, 9974) | 4200 (711, 7689) | 4360 (758, 7963) |
| 3x GDP/capita ($166,845) | 7430 (1570, 13290) | 6028 (905, 11152) | 6188 (979, 11397) |
| ACC, Accident Compensation Corporation; ICER, incremental cost effectiveness ratio; QALY, quality adjusted life year; INB, incremental net benefit; GDP, gross domestic product. * Intervention dominates control (higher QALYs and lower costs). | | | |

## References

1. Bates D, Mächler M, Bolker B, et al. Fitting linear mixed-effects models using lme4. *J Stat Softw* 2015;67(1):1-48. doi: 10.18637/jss.v067.i01

2. Darlow B, Stanley J, Dean S, et al. The Fear Reduction Exercised Early (FREE) approach to low back pain: study protocol for a randomised controlled trial. *Trials* 2017;18(1):484. doi: 10.1186/s13063-017-2225-8

3. White IR, Thompson SG. Adjusting for partially missing baseline measurements in randomized trials. *Stat Med* 2005;24(7):993-1007. doi: 10.1002/sim.1981

4. Jordan K, Dunn KM, Lewis M, et al. A minimal clinically important difference was derived for the Roland-Morris Disability Questionnaire for low back pain. *J Clin Epidemiol* 2006;59(1):45-52. doi: 10.1016/j.jclinepi.2005.03.018

5. Muthu V. The number needed to treat: problems describing non-significant results. *Evid Based Ment Health* 2003;6(3):72-72. doi: 10.1136/ebmh.6.3.72

6. Bender R, Kuss O, Hildebrandt M, et al. Estimating adjusted NNT measures in logistic regression analysis. *Stat Med* 2007;26(30):5586-95. doi: 10.1002/sim.3061

7. Deyo RA, Battie M, Beurskens AJ, et al. Outcome measures for low back pain research. A proposal for standardized use. *Spine* 1998;23(18):2003-13.

8. Lamb SE, Lall R, Hansen Z, et al. A multicentred randomised controlled trial of a primary care-based cognitive behavioural programme for low back pain. The Back Skills Training (BeST) trial. *Health Technol Assess* 2010;14(41):g1-281. doi: 10.3310/hta14410

9. Pocock SJ, Assmann SE, Enos LE, et al. Subgroup analysis, covariate adjustment and baseline comparisons in clinical trial reporting: current practice and problems. *Stat Med* 2002;21(19):2917-30. doi: 10.1002/sim.1296

10. Mannion AF, Wieser S, Elfering A. Association Between Beliefs and Care-Seeking Behavior for Low Back Pain. *Spine* 2013;38(12):1016-25. doi: 10.1097/BRS.0b013e31828473b5

11. Ferreira ML, Machado G, Latimer J, et al. Factors defining care-seeking in low back pain--a meta-analysis of population based surveys. *Eur J Pain* 2010;14(7):747 e1-7. doi: 10.1016/j.ejpain.2009.11.005

12. Hartvigsen J, Hancock MJ, Kongsted A, et al. What low back pain is and why we need to pay attention. *The Lancet* 2018;391(10137):2356-67. doi: 10.1016/S0140-6736(18)30480-X

13. Chou R, Shekelle P. Will this patient develop persistent disabling low back pain? *JAMA* 2010;303(13):1295-302. doi: 10.1001/jama.2010.344

14. Kent P, Mirkhil S, Keating J, et al. The concurrent validity of brief screening questions for anxiety, depression, social isolation, catastrophization, and fear of movement in people with low back pain. *Clin J Pain* 2014;30(6):479-89. doi: 10.1097/ajp.0000000000000010

15. Hart DL, Werneke MW, George SZ, et al. Screening for elevated levels of fear-avoidance beliefs regarding work or physical activities in people receiving outpatient therapy. *Phys Ther* 2009;89(8):770-85. doi: 10.2522/ptj.20080227

16. Nicholas MK, McGuire BE, Asghari A. A 2-item short form of the Pain Self-efficacy Questionnaire: development and psychometric evaluation of PSEQ-2. *J Pain* 2015;16(2):153-63. doi: 10.1016/j.jpain.2014.11.002

17. Wideman TH, Hill JC, Main CJ, et al. Comparing the responsiveness of a brief, multidimensional risk screening tool for back pain to its unidimensional reference standards: the whole is greater than the sum of its parts. *Pain* 2012;153(11):2182-91. doi: 10.1016/j.pain.2012.06.010

18. Shaw WS, Pransky G, Patterson W, et al. Early disability risk factors for low back pain assessed at outpatient occupational health clinics. *Spine* 2005;30(5):572-80. doi: 10.1097/01.brs.0000154628.37515.ef

19. Dionne CE, Bourbonnais R, Frémont P, et al. A clinical return-to-work rule for patients with back pain. *CMAJ* 2005;172(12):1559-67. doi: 10.1503/cmaj.1041159

20. National Ethics Advisory Committee. Ethical guidelines for intervention studies: Revised edition. Wellington, New Zealand: Ministry of Health, 2012.

21. Ioannidis JA, Evans SW, Gøtzsche PC, et al. Better reporting of harms in randomized trials: An extension of the consort statement. *Ann Intern Med* 2004;141(10):781-88. doi: 10.7326/0003-4819-141-10-200411160-00009

22. Lumley T. Analysis of complex survey samples. *J Stat Softw* 2004;9(1):1-19.

23. survey: analysis of complex survey samples [program]. R package version 3.32. version, 2017.

24. Pinto D, Robertson MC, Hansen P, et al. Good agreement between questionnaire and administrative databases for health care use and costs in patients with osteoarthritis. *BMC Med Res Methodol* 2011;11:45. doi: 10.1186/1471-2288-11-45

25. Van Buuren S, Boshuizen HC, Knook DL. Multiple imputation of missing blood pressure covariates in survival analysis. *Stat Med* 1999;18(6):681-94. doi: 10.1002/(SICI)1097-0258(19990330)18:6<681::AID-SIM71>3.0.CO;2-R

26. Van Buuren S, Groothuis-Oudshoorn K. mice: Multivariate imputation by chained equations in R. *J Stat Softw* 2010:1-68.

27. Rubin DB. Multiple imputation after 18+ years. *J Am Stat Assoc* 1996;91(434):473-89.
